# The type 3 secretion effector IpgD promotes *S*. *flexneri* dissemination

**DOI:** 10.1371/journal.ppat.1010324

**Published:** 2022-02-07

**Authors:** Volkan K. Köseoğlu, Marieke K. Jones, Hervé Agaisse

**Affiliations:** 1 Department of Microbiology, Immunology, and Cancer Biology, School of Medicine, University of Virginia, Charlottesville, Virginia, United States of America; 2 Claude Moore Health Sciences Library, University of Virginia, Charlottesville, Virginia, United States of America; University of Toronto, CANADA

## Abstract

The bacterial pathogen *Shigella flexneri* causes 270 million cases of bacillary dysentery worldwide every year, resulting in more than 200,000 deaths. *S*. *flexneri* pathogenic properties rely on its ability to invade epithelial cells and spread from cell to cell within the colonic epithelium. This dissemination process relies on actin-based motility in the cytosol of infected cells and formation of membrane protrusions that project into adjacent cells and resolve into double-membrane vacuoles (DMVs) from which the pathogen escapes, thereby achieving cell-to-cell spread. *S*. *flexneri* dissemination is facilitated by the type 3 secretion system (T3SS) through poorly understood mechanisms. Here, we show that the T3SS effector IpgD facilitates the resolution of membrane protrusions into DMVs during *S*. *flexneri* dissemination. The phosphatidylinositol 4-phosphatase activity of IpgD decreases PtdIns(4,5)P_2_ levels in membrane protrusions, thereby counteracting *de novo* cortical actin formation in protrusions, a process that restricts the resolution of protrusions into DMVs. Finally, using an infant rabbit model of shigellosis, we show that IpgD is required for efficient cell-to-cell spread *in vivo* and contributes to the severity of dysentery.

## Author summary

The intracellular pathogen *Shigella flexneri* is the causative agent of bacillary dysentery (blood in stool). Invasion of epithelial cells and cell-to-cell spread are critical determinants of *S*. *flexneri* pathogenesis. Cell-to-cell spread relies on the formation of membrane protrusions that project into adjacent cells and resolve into vacuoles. The molecular mechanisms supporting this dissemination process are poorly understood. In this study, we show that *S*. *flexneri* employs the phosphatidylinositol phosphatase activity of the T3SS effector protein IpgD to manipulate phosphoinositides in the protrusion membrane. Manipulation of phosphoinositide signaling restricts the formation of actin networks underneath the protrusion membrane, which would otherwise prevent the scission of protrusions into vacuoles. We also demonstrate that IpgD is required for efficient dissemination in the colon of infant rabbits and contributes to the severity of disease. This study exemplifies how manipulation of phosphoinositide signaling by intracellular pathogens supports bacterial pathogenesis.

## Introduction

The intestinal pathogen *Shigella flexneri* is the causative agent of bacillary dysentery [1]. The disease is characterized by a dramatic ulceration of the colonic mucosa, massive infiltration of immune cells, and blood in stool [2]. There are 270 million cases of bacillary dysentery annually in the world, resulting in more than 200,000 deaths, especially in children under age five [3]. Until recently, infected patients were easily cured with antibiotic treatment. However, the isolation of multiple antimicrobial-resistant strains from patients is becoming the norm worldwide [4]. Seminal studies conducted in non-human primates have revealed that bacillary dysentery is associated with bacterial invasion of epithelial cells in the colon [5]. The development of *in vitro* tissue culture systems [6] and genetic approaches [7] led to the discovery that *S*. *flexneri* invasion properties rely on the type 3 secretion system (T3SS) [8], which delivers a panel of effector proteins that manipulate the actin cytoskeleton, leading to bacterial engulfment into primary vacuoles [9]. As they escape primary vacuoles, bacteria exploit the actin cytoskeleton to display actin-based motility in the cytosol of infected cells [10]. *S*. *flexneri* expresses a surface protein, IcsA [11,12], which recruits N-WASP [13], a host cell actin nucleation-promoting factor [14]. N-WASP in turn recruits the ARP2/3 complex, which leads to actin nucleation at the bacterial pole [15]. The forces generated by actin network assembly propel the bacteria throughout the cytosol of infected cells. As motile bacteria encounter cell-cell contact, they form membrane protrusions that project into adjacent cells [16]. The resolution of these protrusions leads to the formation of double-membrane vacuoles [17,18], from which the pathogen escapes by deploying its T3SS and specific effector proteins [19–21].

Although *S*. *flexneri* clearly relies on host factors such as N-WASP and the ARP2/3 complex to display actin-based motility, it has long been unclear whether the formation and resolution of membrane protrusions into DMVs is a mere consequence of actin-based motility or is indeed supported by specific host cell processes. Recently, however, several studies have highlighted the existence of specific mechanisms supporting *S*. *flexneri* cell-to-cell spread beyond the requirement of actin-based motility [22]. The proper formation of protrusions relies on Myosin-X and formins [23,24]. Moreover, the resolution process is dependent on host PI 3-kinase activity and subsequent noncanonical clathrin-mediated endocytosis [25]. Unlike *Listeria monocytogenes*, *S*. *flexneri* protrusions resolve into DMVs via formation of intermediate membrane compartments termed vacuole-like protrusions (VLPs), which requires tyrosine kinase and phosphoinositide signaling pathways [17,18,26]. In addition to host cell factors, the formation of protrusions relies on the T3SS translocase IpaC, whose insertion in the plasma membrane releases membrane tension at cell-cell contacts [27].The T3SS also supports the resolution of membrane protrusions into DMVs [18], through mechanisms that remain to be determined.

Here, we show that the resolution of membrane protrusions is facilitated by the T3SS effector IpgD. IpgD manipulates phosphoinositide signaling in protrusions, which prevents *de novo* formation of cortical actin in membrane protrusions, thereby facilitating the resolution of protrusions into DMVs. We also demonstrate that IpgD contributes to the efficiency of *S*. *flexneri* dissemination and severity of symptoms *in vivo*, using an infant rabbit model of shigellosis [28].

## Results

### The T3SS effector IpgD is required for *S*. *flexneri* dissemination in HT-29 cells

**Fig 1:**
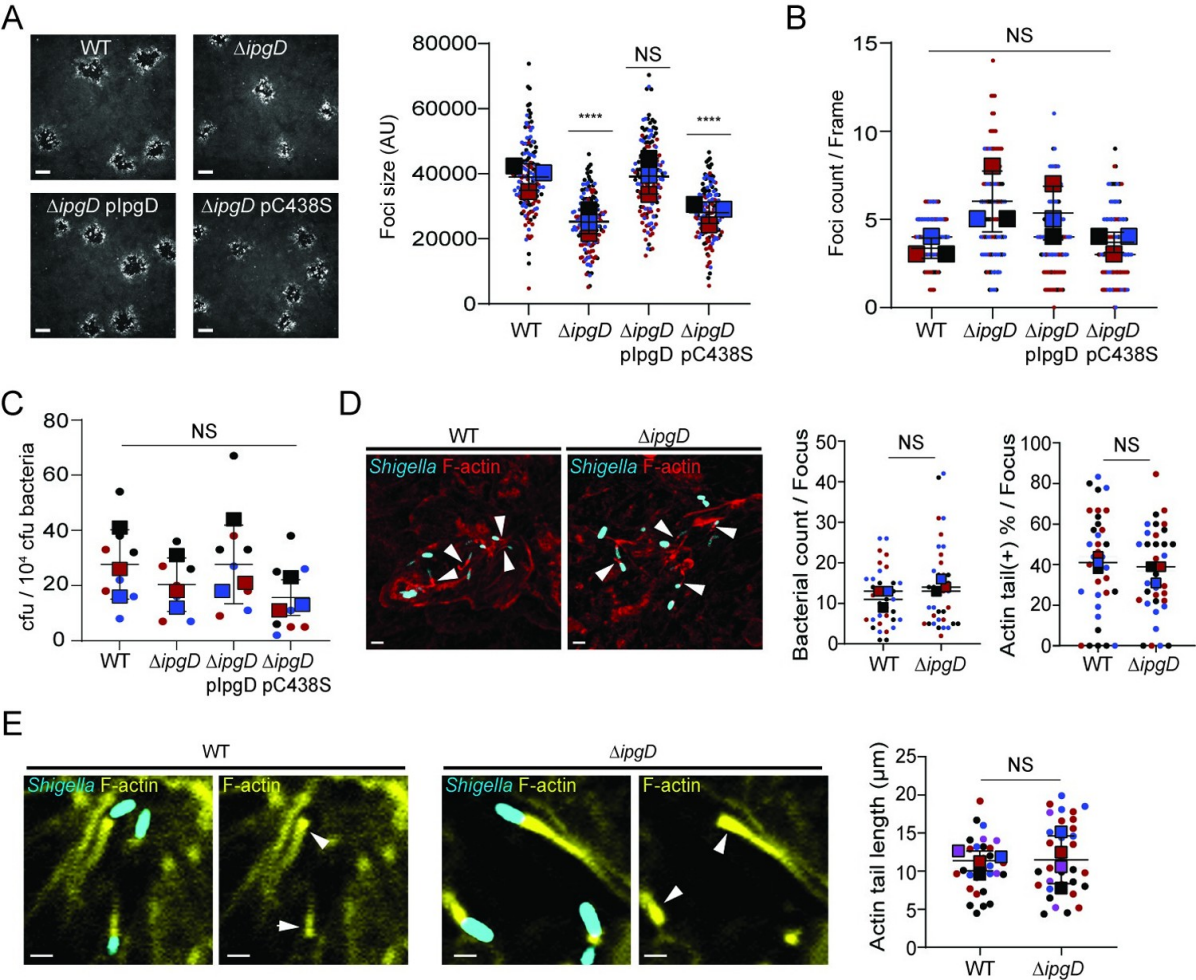
IpgD enzymatic activity promotes the formation of infection foci by *S*. *flexneri* in HT-29 cells. (A) Representative images of infection foci formed in HT-29 cell monolayers infected for 16 h with *S*. *flexneri* strains, wild type (WT), mutant (Δ*ipgD*), mutant expressing wild type IpgD (pIpgD) and enzymatically inactive IpgD mutant (pC438S). Scale bar is 30 μm. Graph showing infection foci size (area) in arbitrary units (AU). Squares represent means of 150 foci for each strain with s.d. (*n = 3*) (B) Number of infection foci formed by *S*. *flexneri* strains in HT-29 cell monolayers after 16 hpi (corresponding to the experiments in Panel A). Squares represent means of foci number per frame with s.d. (120 frames per strain, *n = 3*). (C) Cell invasion by *S*. *flexneri* strains determined with gentamicin protection assay. Squares represent means of colony forming unit (cfu) with s.d. per 10000 cfu bacteria used for invasion (*n = 3*). (D) Representative images of WT pCFP and Δ*ipgD* pCFP infection focus. Blue, bacteria; red, F-actin (Phalloidin-594); white arrowheads, actin tails; scale bar, 5 μm. Graphs showing the bacterial counts and the ratio of bacteria displaying actin tails in each infection focus 4 hpi. Squares represent means of bacterial count and ratio of bacteria with actin tails (Actin tail(+)) from 35 WT pCFP and 36 Δ*ipgD* pCFP infection foci with s.d. (*n* = 3). (E) Representative images showing actin tails of intracellular *S*. *flexneri* pCFP (WT and Δ*ipgD*). Blue, bacteria; yellow, F-actin (Phalloidin-514); white arrowheads, actin tails; scale bar, 2 μm. Graph showing lengths of actin tails formed by WT and Δ*ipgD* strains. Squares represent means of 30 WT pCFP and 33 Δ*ipgD* pCFP actin tail lengths with s.d. (*n* = 4). Circles represent data points and colors indicate independent biological replicate groups (A-E). Statistics: Panels A-C, one-way ANOVA followed by Tukey’s multiple comparisons (WT, control); Panel D and E, unpaired t-test analysis; ****P < 0.0001; NS, not significant.

To address the role of the T3SS in *S*. *flexneri* dissemination, we investigated a collection of T3SS effector mutants for their ability to spread from cell to cell. We identified *ipgD* as a potential candidate whose deletion caused 35% reduction in the size of the infection foci formed in HT-29 cells (Fig 1A). *In vitro* and *in vivo* studies have shown that IpgD is a phosphatidylinositol 4-phosphatase [29]. To determine whether the phosphatidylinositol 4-phosphatase activity of IpgD is required for efficient cell-to-cell spread, we compared the size of the infection foci formed in HT-29 cells infected with the Δ*ipgD* mutant expressing wild type (pIpgD) or catalytically dead IpgD (pIpgD^C438S^). The spreading defect observed in cells infected with the Δ*ipgD* mutant was complemented by expression of wild type IpgD, but not by expression of IpgD^C438S^ (Fig 1A). These results suggest that the phosphatidylinositol 4-phosphatase activity of IpgD is required for *S*. *flexneri* spread from cell to cell.

IpgD was characterized as a T3SS effector protein that supports the formation of large membrane ruffles at sites of invasion in HeLa cells [30]. The role of IpgD in the invasion process *per se* is however controversial. Initial studies reported that the IpgD mutant was as invasive as the isogenic M90T parental strain [31]. However, recent studies using the 2457T strain reported a role for IpgD in invasion of HeLa cells through ARF6 activation [32]. In HT-29 cells, the Δ*ipgD* strain was as invasive as the isogenic 2457T parental strain, as determined by infection foci counts (Fig 1B) and gentamicin protection assay (Fig 1C).

In HeLa cells, an *ipgD* mutant was reported to exhibit a delayed escape from primary vacuoles within 30 min after invasion [33].To assess whether a delayed escape from primary vacuoles could account for the apparent cell-to-cell spreading defect displayed by the Δ*ipgD* strain (Fig 1A), we quantified the total number of bacteria per infection focus and the proportion of bacteria displaying actin tails 4 hours post-infection (hpi), as a readout of cytosolic bacteria. We observed similar bacterial counts and proportions of actin tails in cells infected with the wild type and Δ*ipgD* strains (Fig 1D). Furthermore, the wild type and Δ*ipgD* strains formed actin tails of similar length (Fig 1E), indicating similar cytosolic motility, since the length of actin tails correlates with the speed of cytosolic bacteria [34]. Collectively, these data suggest that, in HT-29 cells, (i) the Δ*ipgD* strain is as invasive as the wild type strain, (ii) delayed vacuole escape, if any, does not impact the number of cytosolic bacteria, and (iii) cytosolic bacteria display normal actin tails 4 hpi. These results therefore suggest that the impaired cell-to-cell spread of the Δ*ipgD* strain probably originates from a defect in a subsequent step during the dissemination process, including membrane protrusion formation/resolution, and/or DMV escape.

### IpgD supports membrane protrusion resolution during *S*. *flexneri* cell-to-cell spread

**Fig 2:**
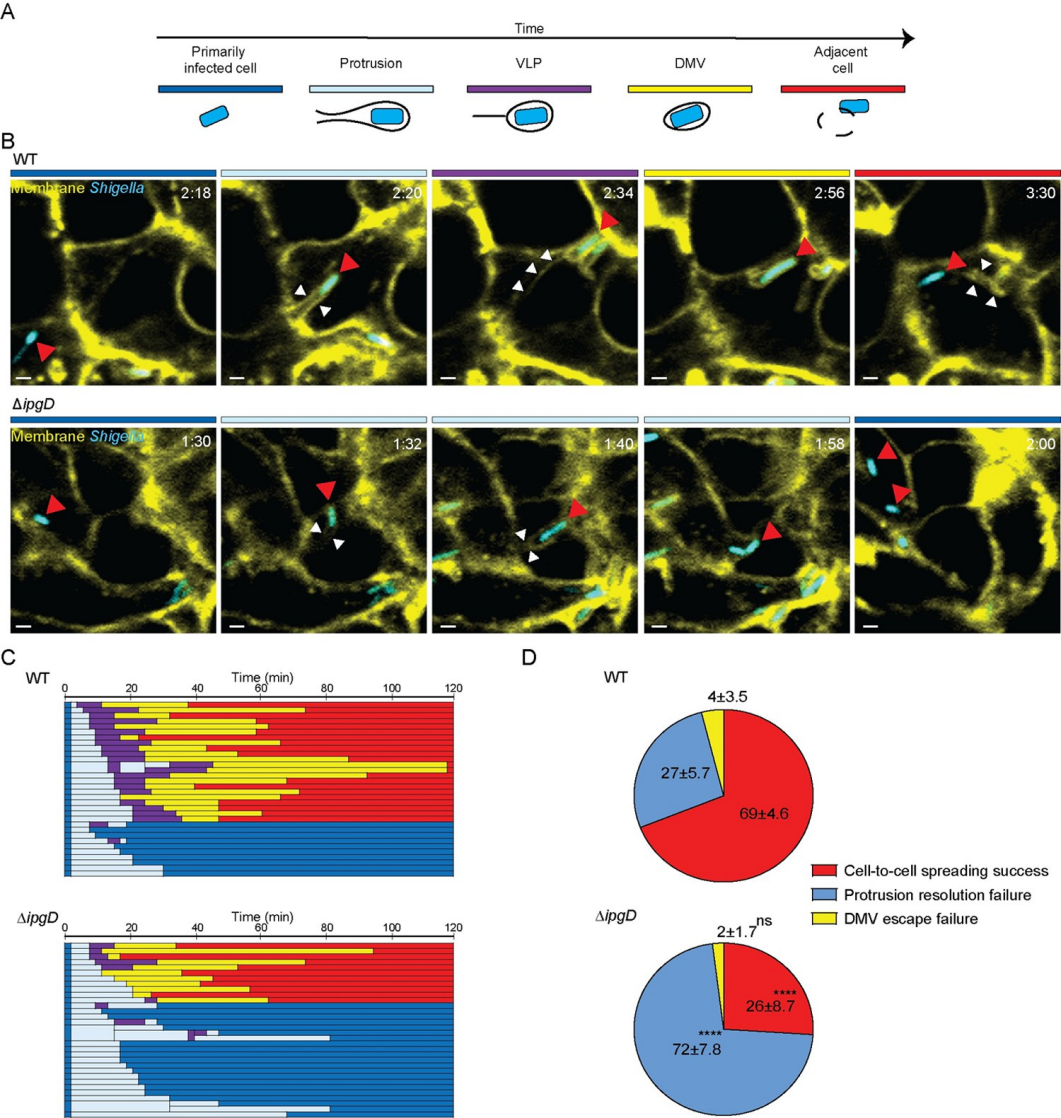
The Δ*ipgD* mutant displays defective protrusion resolution during cell-to-cell spread. (A) Schematic and color-coded representation of the compartments occupied by *S*. *flexneri* during dissemination: dark blue, primarily infected cells; light blue, protrusions; purple, vacuole-like protrusions (VLPs); yellow, double membrane vacuoles (DMVs) and red, cytosol of adjacent cells. *S*. *flexneri* is depicted in blue; the black lines represent the plasma membrane surrounding the bacteria. (B) Representative images showing successful (WT) and failed (Δ*ipgD*) cell-to-cell spread. During successful dissemination, WT pCFP strain displays the formation of canonical membrane compartments (red arrows), including protrusion (t[2:20]; white arrowheads, protrusion neck), VLP (t[2:34]; white arrowheads, membrane tether of VLP), DMV (t[2:56]) and vacuolar escape (t[3:30]; white arrowheads, DMV remnants). Upon failed dissemination due to protrusion resolution failure, Δ*ipgD* pCFP strain forms protrusion (t[1:32]; white arrowheads, protrusion neck) that does not transition into VLP (t[1:32–1:58]; white arrowheads, protrusion neck) ultimately collapses back to the primarily infected cell (t[2:00]). Color-coded bars above images, membrane compartment represented in Panel A; t, relative time as hour: minute; scale bar, 2 μm. (C) Tracking data from one of the independent biological replicates (*n* = 3). At least thirty bacteria were tracked in each biological replicate. The length of each bar reflects the time spent (min) in each color-coded compartment (D) Pie chart showing the proportion of WT and Δ*ipgD* successful cell-to-cell spread (red), protrusion resolution failure (blue), and DMV escape failure (yellow) as mean (+/- s.d.) of all biological replicates (*n =* 3). Statistics: two-way ANOVA statistical analysis followed by Sidak’s multiple comparison test comparing strains; ****P< 0.0001; NS, not significant.

Our previous work revealed that *S*. *flexneri* spreads from cell to cell through the sequential formation of protrusions, vacuole-like protrusions (VLPs) and double-membrane vacuoles (DMVs), from which the pathogen escapes to gain access to the cytosol of adjacent cells [17] (Fig 2A). To determine which dissemination step(s) may be compromised in cells infected with the Δ*ipgD* strain, we conducted time-lapse confocal microscopy of HT-29 cells expressing plasma membrane-targeted yellow fluorescent protein (mbYFP) infected with CFP-expressing *S*. *flexneri*. Tracking results showed that the majority of wild type bacteria (69%) successfully spread to adjacent cells through sequential formation of protrusions, VLPs and DMVs, and proceeded to DMV escape (Figs 2B–2D and S1 and S1 Movie). In stark contrast, the majority of Δ*ipgD* bacteria (72%) failed to spread to adjacent cells (Fig 2D). Although Δ*ipgD* bacteria were able to form protrusions, the majority of the formed protrusions did not transition into the VLP stage, and ultimately collapsed back to primarily infected cells (Figs 2B–2D and S1 and S2 Movie). In the formed protrusions, the Δ*ipgD* strain recruited comparable levels of ARP2/3 at the bacterial pole, suggesting that the Δ*ipgD* mutant displayed normal actin-based motility in protrusions (S2 Fig). We noted that the reliance on IpgD for efficient spread was not absolute, and 26% of the bacteria successfully spread (Figs 2C and 2D and S1), suggesting the existence of redundancy and potential alternative mechanisms in this system. In those instances where the Δ*ipgD* mutant formed DMVs, the time spent in DMV until escape was similar to wild type strain, showing that IpgD is not required for DMV escape (S3 Fig). In addition, wild type and mutant strains exhibited no difference in the time spent in protrusions until successful protrusion resolution or protrusion collapse (S3 Fig). Altogether, these results suggest that IpgD is specifically required for the step of protrusion resolution during *S*. *flexneri* spread from cell to cell.

### PtdIns(4,5)P_2_ clearance correlates with protrusion resolution success

**Fig 3:**
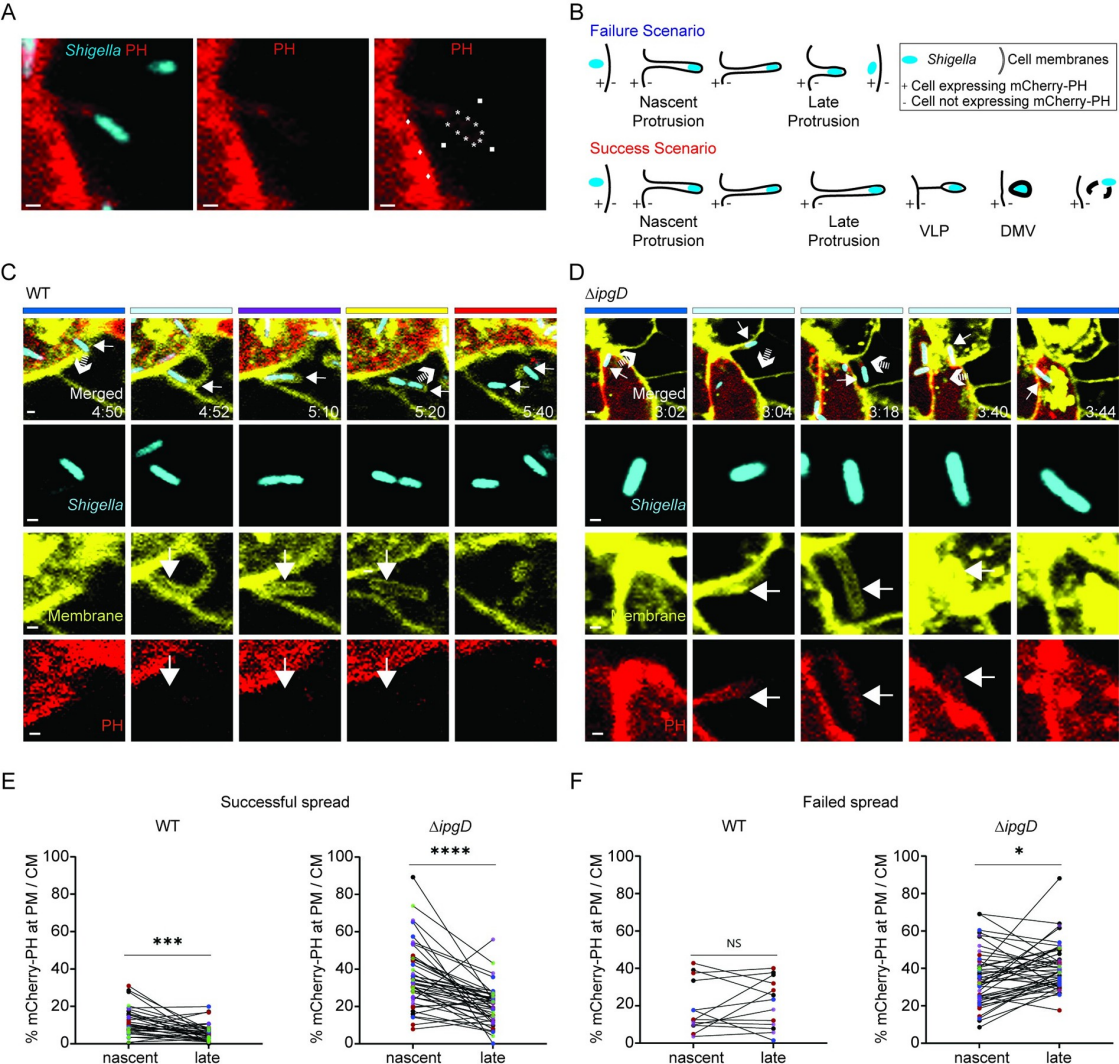
Dynamics of PtdIns(4,5)P_2_ levels in protrusion membrane during *S*. *flexneri* cell-to-cell spread. (A) Quantification of mCherry-PH probe at protrusion membrane. Representative images showing *S*. *flexneri* pCFP (WT) protrusion projecting from a mCherry-PH(+) cell into a mCherry-PH(-) cell. Blue, *Shigella*; red, mCherry-PH. Quantified signals; white stars, protrusion membrane; white diamonds, plasma membrane at cell-cell contact; white squares, local background in mCherry-PH-negative cell. Calculation: mean signal values at protrusion membrane (white stars) and cell membrane (white diamonds) were corrected by subtracting mean background signal value (white squares), then corrected protrusion membrane mCherry-PH signal was divided by corrected cell membrane mCherry-PH signal which is expressed as percentage (%). Scale bar, 1 μm. (B) Graphical representation of tracking showing failed spread (Failure Scenario, blue) and successful spread (Success Scenario, red) from mCherry-PH(+) cell to mCherry-PH(-) cell. Protrusion states (nascent and late) at which mCherry-PH signals were quantified for each scenario. Nascent protrusion refers to the newly formed protrusion for both scenarios. Late protrusion refers to the protrusion preceding collapse (failure scenario) or VLP formation (success scenario). (C) Representative images of successful dissemination. WT *S*. *flexneri* pCFP (blue, small white arrow, merged panel) forming a membrane protrusion (t[4:52]; large white arrow, plasma membrane, yellow) devoid of mCherry-PH probe (t[4:52]; large white arrow, PH, red) that successfully resolves into a DMV (t[5:20]; large white arrow, mCherry-PH, red) from which the bacterium escapes (t[5:40]; small white arrow in merged panel). (D) Representative images of failed dissemination. Δ*ipgD S*. *flexneri* pCFP (blue, small white arrows in merged panel) forming a membrane protrusions (t[3:04]; large white arrow, plasma membrane, yellow) harboring mCherry-PH probe (t[3:04], t[3:18], and t[3:40]; large white arrow, mCherry-PH probe, red) that fails to resolve into a DMV and collapses back to the primary infected cell (t[3:40] and t[3:44]; merged panel, small arrow). Color-coded bars on top indicate the compartments occupied by the bacteria. t, relative time, hour: minute; scale bar, 2 μm for merged panel, 1 μm for single color. White arrows with striped tails in Merged panel images indicate the direction of bacterial movement and predict *S*. *flexneri* location in the next frame. (E-F) Graphs showing how mCherry-PH probe levels (%) change between nascent protrusion membrane and late protrusion membrane during successful (E) and failed (F) *S*. *flexneri* pCFP (WT and Δ*ipgD*) spread (PM, Protrusion Membrane; CM, Cell Membrane). Circles represent data points and colors indicate independent biological replicate groups; 51 mCherry-PH measurements, WT pCFP protrusions (nascent, late); 86 mCherry-PH measurements, Δ*ipgD* pCFP protrusions (nascent, late); (*n* = 5). Statistics: paired t-test analysis; ****P<0.0001, ***P<0.001, *P<0.05; NS, not significant.

Since our previous work showed a role for the T3SS in protrusion resolution [18] and previous studies showed that the T3SS effector protein IpgD hydrolyzes PtdIns(4,5)P_2_ [29], we hypothesized that the regulation of PtdIns(4,5)P_2_ levels in protrusions is critical for protrusion resolution. To test this hypothesis, we generated a HT-29 mbYFP cell line stably expressing mCherry fused to the PH domain of PLCδ (herein referred to as the mCherry-PH probe), which specifically binds PtdIns(4,5)P_2_ [35,36]. We conducted time-lapse confocal imaging experiments using CFP-expressing bacteria and mixed populations of HT-29 mbYFP/ mCherry-PH and HT-29 mbYFP cells. We used computer-assisted image analysis to quantify the signal levels corresponding to the mCherry-PH probe at the plasma membrane surrounding *S*. *flexneri* in protrusions projecting from HT-29 mbYFP/mCherry-PH-positive cells into HT-29 mbYFP-positive/mCherry-PH-negative cells (Fig 3A, white stars). Probe levels at protrusion membranes were corrected for local background (Fig 3A, white squares) and normalized with respect to the probe signal at cell-cell contact (Fig 3A, white diamonds), to account for cell-to-cell variation in probe expression levels. Tracking protrusions using the mbYFP probe allowed us to determine whether a given protrusion would fail to resolve into a DMV and collapse (Fig 3B, failure scenario), or successfully resolve into a DMV (Fig 3B, success scenario). Probe levels were quantified when a new protrusion was formed (Fig 3B, nascent protrusion) and right before the elongated protrusion either collapsed back to the primarily infected cell (Fig 3B, failure scenario, late protrusion) or became a VLP in the adjacent cell (Fig 3B, success scenario, late protrusion). The approach revealed a statistically significant (WT, P = 0.007; Δ*ipgD*, P<0.0001) correlation between successful protrusion resolution and decreased probe levels in late protrusions with respect to nascent protrusions (Fig 3C and 3E, successful spread). Conversely, protrusion resolution failure correlated with similar (Fig 3F, failed spread, wild type) or increased (Fig 3D and 3F, failed spread, Δ*ipgD*) levels of the mCherry-PH probe in late protrusions compared to nascent protrusions. The expression of wild-type IpgD (pIpgD) in Δ*ipgD* completely restored successful protrusion resolution (S4A Fig), which correlated with reduced mCherry-PH probe levels in late protrusions compared to nascent protrusions (S4B Fig, Protrusion resolution). However, the expression of catalytically dead pIpgD^C438S^ did not rescue Δ*ipgD* protrusion resolution defect (S4A Fig), which correlated with increased mCherry-PH probe levels in late protrusions compared to nascent protrusions (S4B Fig, Protrusion collapse, Δ*ipgD* pIpgD::C438S). Together with the observation that the vast majority of the protrusions fail to resolve into DMVs in cells infected with the Δ*ipgD* strain (Fig 2), these results indicate that IpgD-mediated clearance of PtdIns(4,5)P_2_ is critical for protrusion resolution. We however note that PtdIns(4,5)P_2_ clearance occasionally occurs in the protrusions formed in cells infected with Δ*ipgD* (Fig 3E, successful spread) and Δ*ipgD* pIpgD::C438S strains (S4B Fig, Protrusion resolution) through unknown mechanism(s) that may involve uncharacterized bacterial or host cell factors.

### *de novo* cortical actin formation correlates with protrusion resolution failure

**Fig 4:**
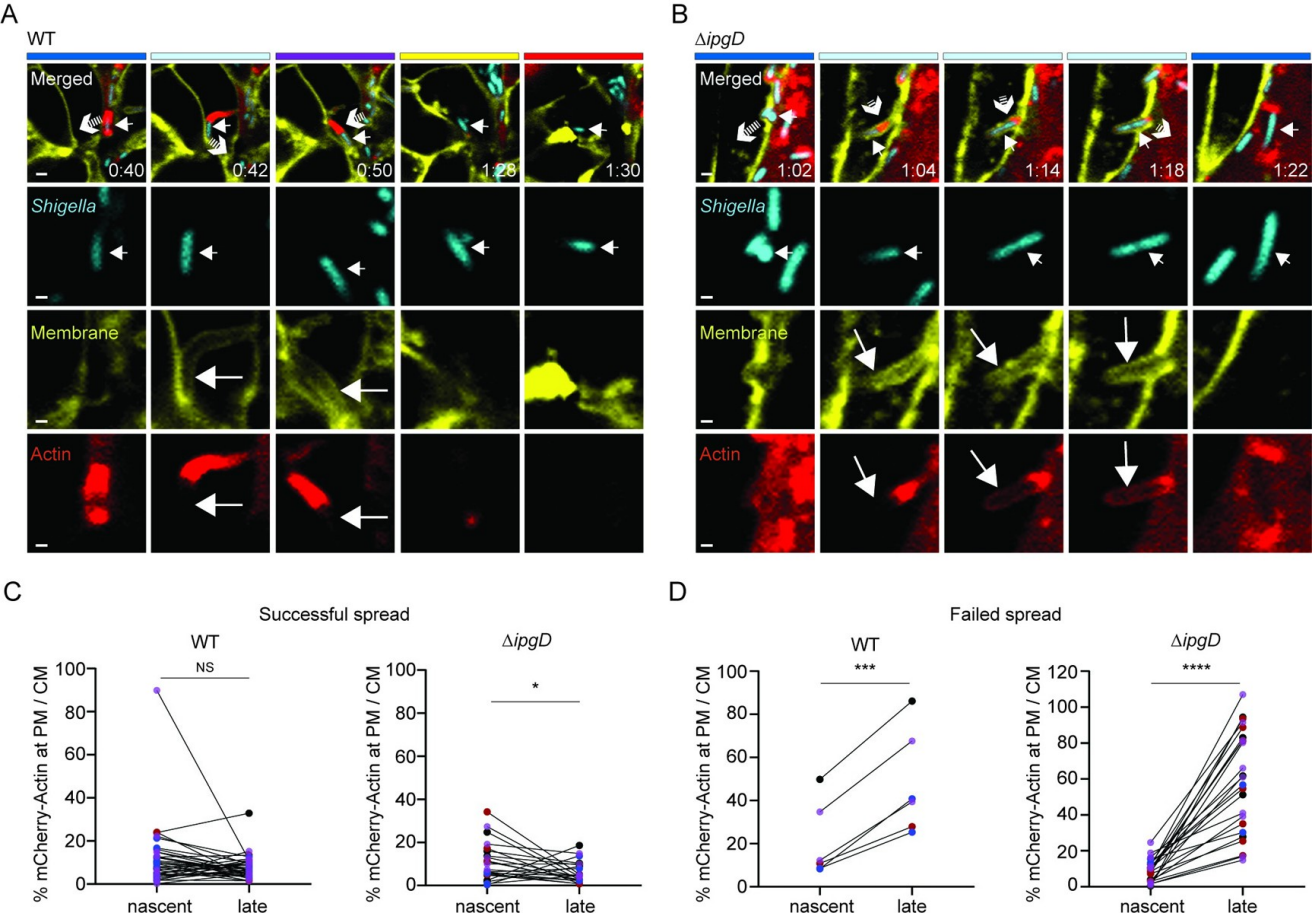
Dynamics of cortical actin levels in protrusion membrane during *S*. *flexneri* cell-to-cell spread. (A) Representative images for successful dissemination. WT *S*. *flexneri* pCFP (blue, small white arrow, merged panel) forming a membrane protrusion (t[0:42]; large white arrow pointing to plasma membrane, yellow) exhibiting very low/undetectable cortical actin (large white arrow, mCherry-Actin, red) that successfully resolves into a DMV (t[1:28]) from which the bacterium escapes (t[1:30]; small white arrow, merged panel). (B) Representative images for failed dissemination. Δ*ipgD S*. *flexneri* pCFP (blue, small white arrow, merged panel) forming a membrane protrusion (t[1:04]; large white arrow pointing to plasma membrane, yellow) that starts displaying cortical actin at later time point (t[1:14]; large white arrow, mCherry-Actin, red), fails to resolve into a DMV and collapses back to the primarily infected cell (t[1:22]; small white arrow, merged panel). Color-coded bars on top indicate the compartments occupied by the bacteria. t, relative time, hour: minute; scale bar, 2 μm for merged panel, 1 μm for single color. White arrows with striped tails in Merged panel images indicate the direction of bacterial movement and predict *S*. *flexneri* location in the next frame. (C-D) Graphs showing how mCherry-Actin probe levels change between nascent protrusion membrane and late protrusion membrane during successful (C) and failed (D) *S*. *flexneri* pCFP (WT and Δ*ipgD*) spread; mCherry-Actin levels (%) at protrusion membrane (PM) normalized with mCherry-Actin levels at cell membrane (CM) as described in Fig 3. Circles represent data points and colors indicate replicate groups; 47 mCherry-Actin measurements, WT pCFP and Δ*ipgD* pCFP protrusions (nascent, late); (*n* = 4). Statistics: paired t test analysis; ****P<0.0001, ***P<0.001, *P<0.05; NS, not significant.

PtdIns(4,5)P_2_ levels play a critical role in the assembly and dynamics of actin networks at the plasma membrane [37,38], herein referred to as cortical actin. Cortical actin is a critical determinant of cell shape. However, in the context of cell-to-cell spread, the presence of cortical actin in protrusions may impede protrusion scission into DMVs, leading to defective cell-to-cell spread. To investigate cortical actin levels during *S*. *flexneri* cell-to-cell spread, we generated a HT-29 mbYFP cell line expressing β-actin fused to mCherry (mCherry-Actin). As expected, the probe accumulated at the bacterial pole and in the protrusion neck, reflecting actin-based motility in protrusions. Importantly, we also observed cortical actin underneath the plasma membrane surrounding the bacteria in protrusions (S5 Fig). Correlative analysis on fixed samples demonstrated that the probe mainly reports on polymerized actin (S5 Fig, regression plot). To determine the dynamics of cortical actin in protrusions, we conducted time-lapse confocal imaging to quantify changes in mCherry-Actin levels at protrusion plasma membranes surrounding CFP-expressing bacteria, where cortical actin visualization is not obscured by the actin tails generated at the bacterial pole in the neck of protrusions. We applied the same quantification method described for mCherry-PH probe (Fig 3A and 3B) to determine changes in mCherry-Actin levels. All nascent protrusions displayed low cortical actin levels (Fig 4A and 4B). At later time points, we observed situations in which low levels of cortical actin were maintained (Fig 4A, WT, t [0.42–0:50]) and situations showing *de novo* cortical actin formation (Fig 4B, Δ*ipgD*, t[1:14–1:18]). Importantly, tracking each protrusion demonstrated that low cortical actin at late protrusions correlated with successful protrusion resolution by the Δ*ipgD* strain (Fig 4C, Successful spread, Δ*ipgD*). Conversely, *de novo* cortical actin formation correlated with protrusion resolution failure with both strains (Fig 4D, Failed spread and S3 Movie). These results show that the formation of nascent protrusions occurs as motile bacteria pierce through the existing cortical actin at sites of cell-cell contact, leading to deformation of the plasma membrane into a low cortical actin membrane-bound compartment that protrudes into adjacent cells. As protrusions elongate, IpgD confers to the bacteria the ability to restrict *de novo* cortical actin formation, which facilitates the resolution of protrusions into DMVs.

### PtdIns(4,5)P_2_ levels correlates with cortical actin levels

**Fig 5:**
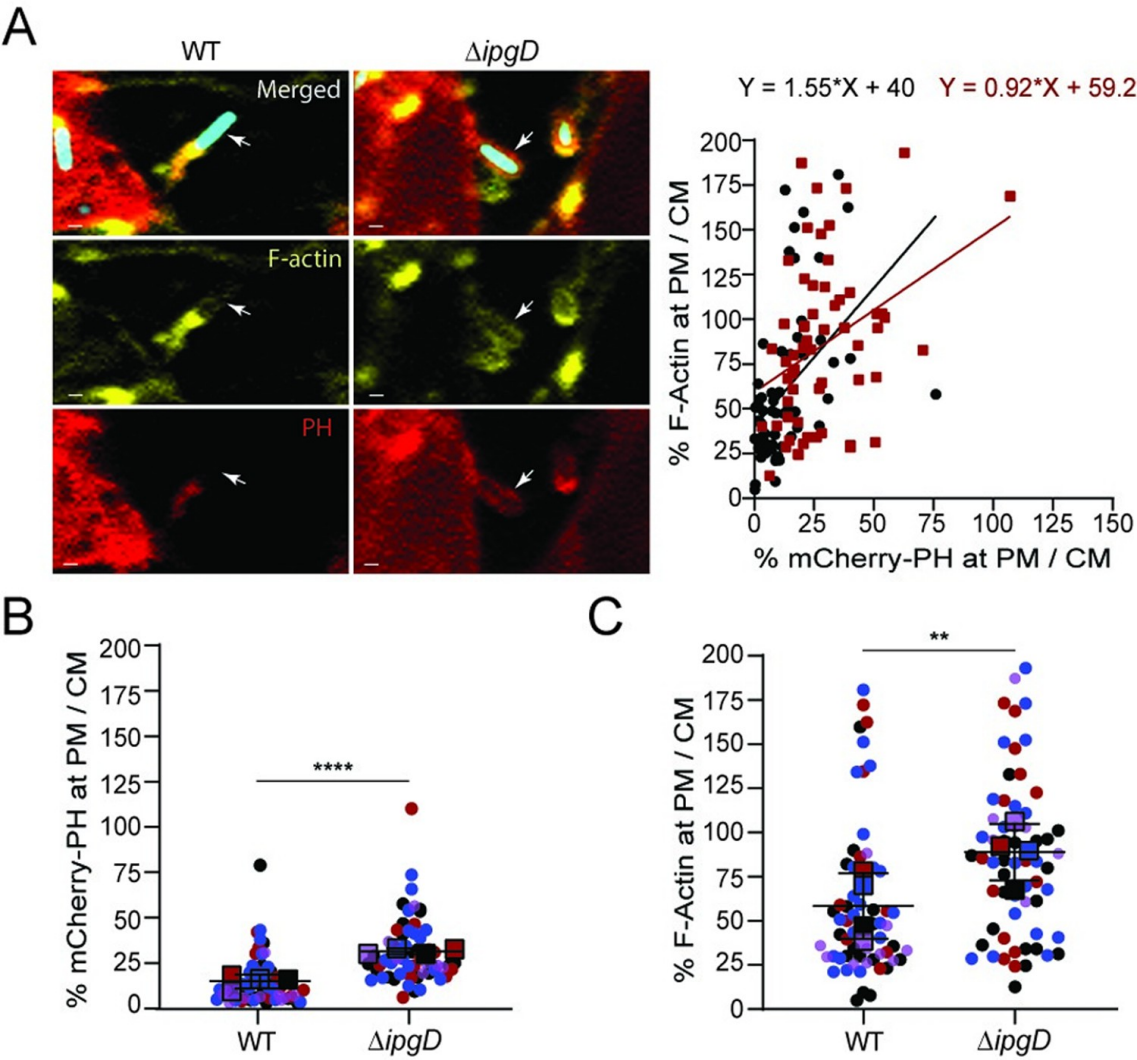
PtdIns(4,5)P_2_ levels correlate with cortical actin levels. (A) Representative images of WT pCFP and Δ*ipgD* pCFP protrusions showing colocalization of F-actin and mCherry-PH probe. Blue, *Shigella*; white arrows: F-actin (Phalloding-514, yellow) and mCherry-PH probe (PH, red); scale bar, 1 μm. Linear regression plot showing F-actin and mCherry-PH signals at protrusion membrane (PM) normalized with corresponding signals at cell membrane (CM) (quantification described in S6 Fig); 62 WT pCFP protrusions, 61 Δ*ipgD* pCFP protrusions; black circles, WT pCFP protrusions; red squares, Δ*ipgD* pCFP protrusions. Statistics: significance of mCherry-PH and F-actin relationship is determined by the difference of slope from zero. **P<0.01 based on a linear regression analysis explaining F-actin by mCherry-PH, strain, and an interaction between mCherry-PH and strain (R^2^ = 0.22, F_3,119_ = 13.02, P<0.0001). Slopes of WT pCFP and Δ*ipgD* pCFP are not statistically different (P = 0.198). (B) Graph showing mCherry-PH levels at protrusion membrane (PM) normalized with mCherry-PH levels at cell membrane (CM); (quantification described in S6 Fig). (C) Graph showing F-actin at protrusion membrane (PM) normalized with F-actin levels at cell membrane (CM); (quantification described in S6 Fig). (B-C) Circles represent data points and colors indicate independent biological replicate groups; means (squares) of measurement from four biological replicates with s.d.; 62 WT pCFP protrusions and 61 Δ*ipgD* pCFP protrusions; (*n* = 4). Statistics: unpaired t-test, ****P<0.0001, **P<0.01.

To further establish the correlation between the regulation of PtdIns(4,5)P_2_ levels and *de novo* cortical actin formation in protrusions, we carried out infection experiments with CFP-expressing *S*. *flexneri* strains and monolayers of mixed HT-29 mbCFP/ mCherry-PH and HT-29 mbCFP cells. Using fixed samples, we assessed the co-localization of mCherry-PH probe with F-actin underneath the plasma membrane surrounding bacteria in protrusions (S6 Fig). The approach demonstrated a statistically significant relationship between mCherry-PH probe and F-actin signal levels for both strains (Fig 5A, regression plot, P<0.0001), showing that high levels of PtdIns(4,5)P_2_ correlate with high levels of cortical actin in membrane protrusions. In support of the notion that cortical actin restricts the spread of the *ΔipgD* strain, we detected significantly higher mCherry-PH and F-actin levels in protrusions formed by the *ΔipgD* strain compared to wild type bacteria (Fig 5B and 5C). Collectively, these results support the notion that the 4-phosphatidylinositol phosphatase activity of IpgD decreases the levels of PtdIns(4,5)P_2_ in protrusions, which prevents *de novo* cortical actin formation, and facilitates the resolution of protrusions into vacuoles.

### IpgD is required for efficient cell-to-cell spread *in vivo*

**Fig 6:**
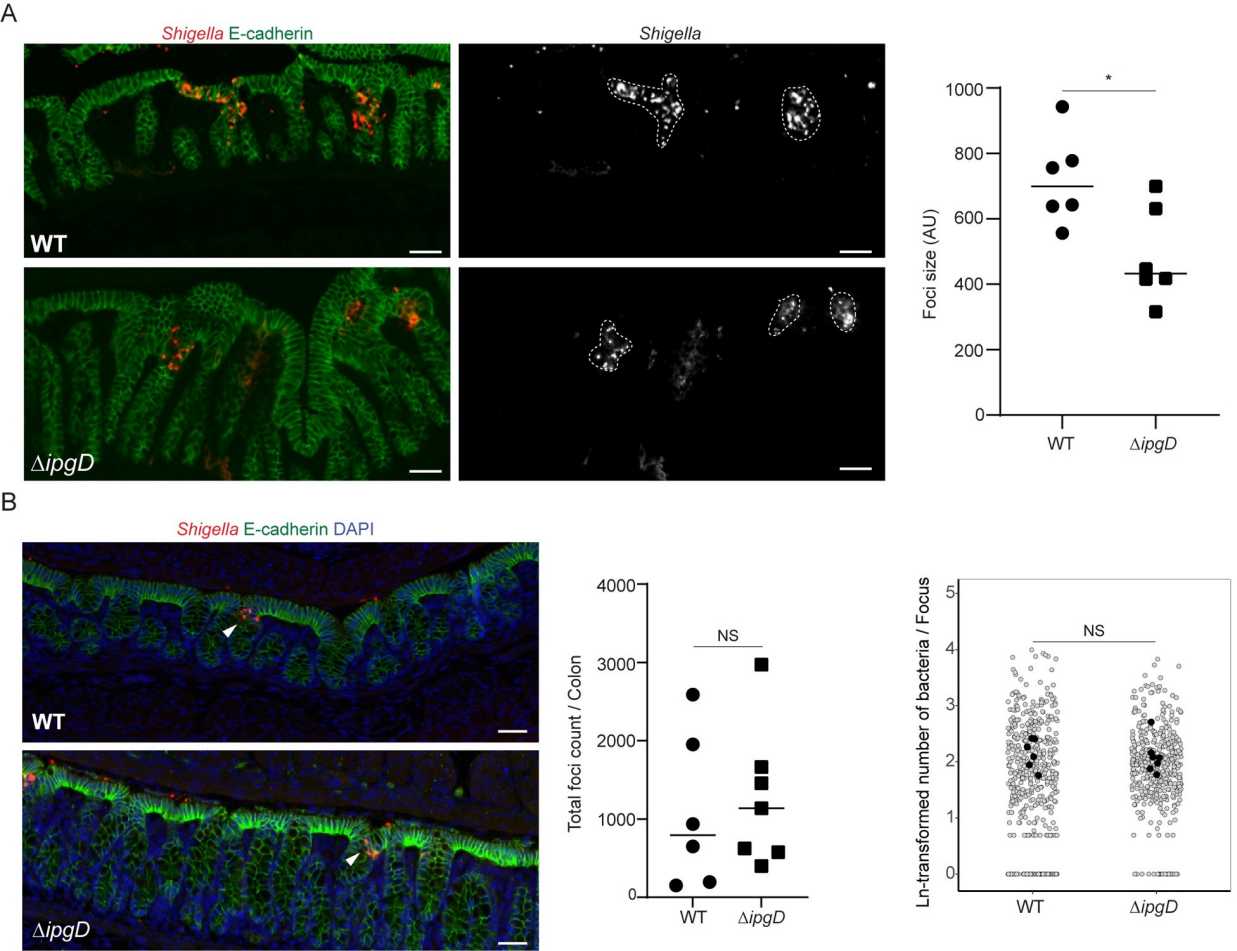
IpgD is required for efficient cell-to-cell spread *in vivo*. (A) Representative images of infant rabbit colon sections showing infection foci formed by *S*. *flexneri* (red in merged, white in *Shigella* panel; WT pCFP and Δ*ipgD* pCFP) in E-cadherin-positive epithelial cells (green) 8 hpi. Dashed lines, infection foci; scale bar, 20 μm. Graph showing average of infection foci sizes (AU, area in arbitrary units) observed for each animal (*n* = 6 per strain; WT pCFP, 130 infection foci; Δ*ipgD* pCFP, 88 infection foci). Statistics: unpaired t-test, *P<0.05. (B) Representative images of infant rabbit colon sections showing invaded regions (white arrowheads) by *S*. *flexneri* pCFP (red, WT and Δ*ipgD*) in E-cadherin-positive epithelial cells (green) 4 hpi; DAPI staining (blue); scale bar, 30 μm. Graph on the left showing total number of infection foci per entire colon (WT-infected colons, 6; Δ*ipgD*-infected colons, 7). Statistics: unpaired t-test; NS, not significant. Graph on the right showing number of bacteria in natural logarithmic scale (Ln) at each infection focus. Black circles represent means of measurements from all frames of colon sections and gray circles represent individual measurements from each frame of colon sections. Statistics: linear mixed model analysis; NS, not significant.

Recent studies have established that many features of human shigellosis, including epithelial cell invasion and cell-to-cell spread, can be recapitulated in the colon of infant rabbits.[28,39]. To determine the role of IpgD in dissemination *in vivo*, we compared the size of infection foci formed by wild type and Δ*ipgD* strains in the colon of infant rabbits 8 hpi. Similar to the results obtained in HT-29 cells (Fig 1), the Δ*ipgD* strain formed significantly smaller infection foci compared to the wild type strain (Fig 6A). We also confirmed that IpgD is not essential for bacterial invasion, as the numbers of infection foci per colon and the number of bacteria per infection focus were comparable for wild type and Δ*ipgD* strains 4 hpi (Fig 6B, detail quantification method in S7 Fig). We also investigated a potential role of IpgD in nonphagocytic cell apoptosis [40,41].To this end, we compared cell death as assessed by TUNEL assay in the colon of animals infected with wild type and Δ*ipgD* strains. We observed no differences in the rate of cell death in either un-infected or infected regions, regardless of the bacterial strains used for infection (S8 Fig), suggesting that cell death is probably not the cause of the cell-to-cell spreading defect observed in animals infected with the Δ*ipgD* strain. Collectively, these results show that, similar to the results obtained in HT-29 cells, IpgD promotes cell-to-cell spread *in vivo*.

### Role of IpgD in pathogenesis

**Fig 7:**
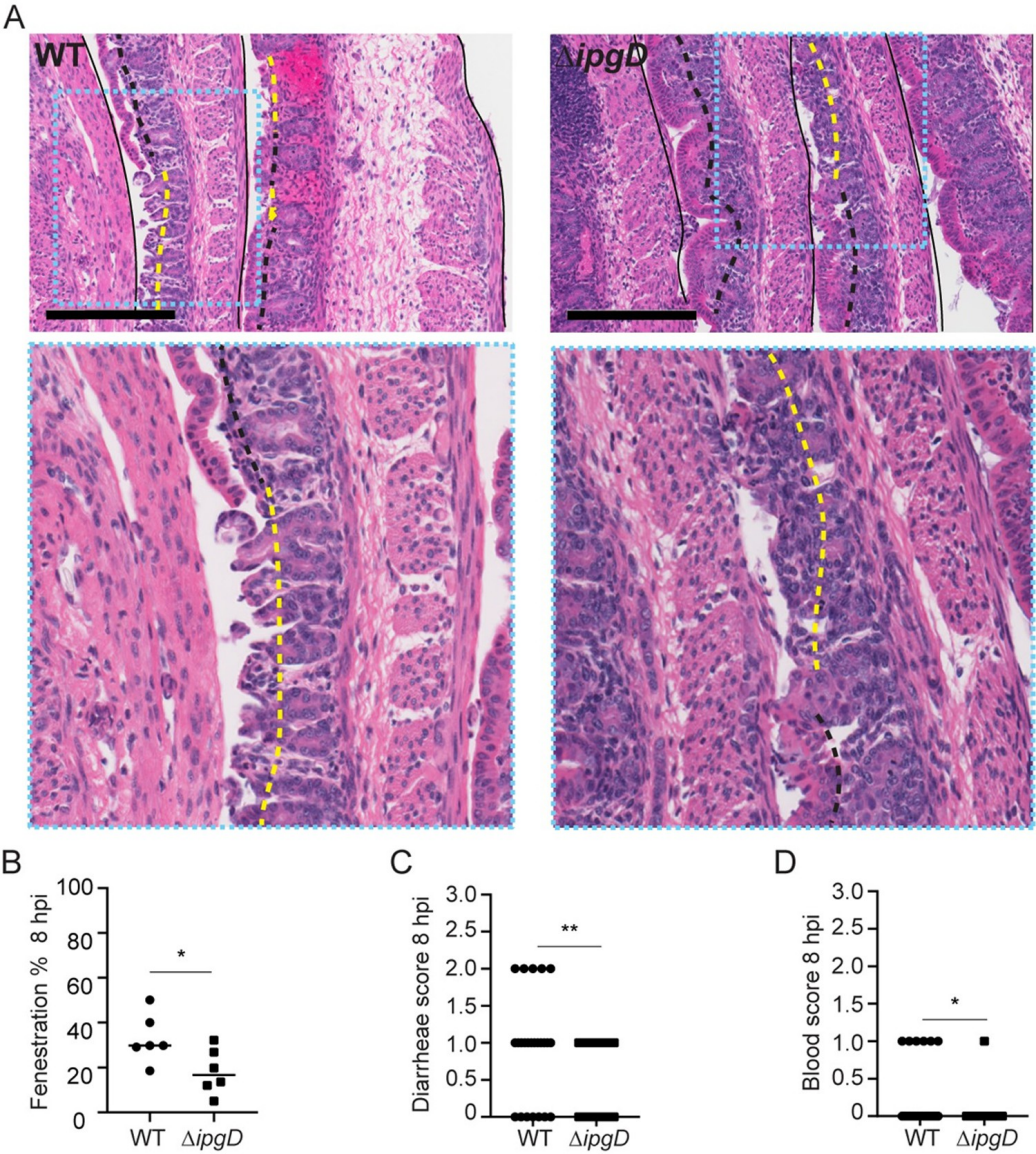
Role of IpgD in pathogenesis. (A) Representative images of hematoxylin- and eosin-stained colonic sections from animals infected with the *S*. *flexneri* pCFP (WT and Δ*ipgD*) 8 hpi. Intact black lines, colonic tissues; dashed black lines, intact epithelium; yellow dashed lines, fenestrated epithelium; scale bar, 200 μm; inset, turquoise dashed rectangle, magnified colon regions to illustrate intact and fenestrated epithelium. (B) Graph showing quantification of colon fenestration at 8 hpi (*n* = 6 per strain). (C) Graph showing diarrhea scores of 24 animals per strain 8 hpi. (D) Graph showing blood scores of 24 animals per strain 8 hpi. Statistics (Panels B-D): unpaired t-test; *P<0.05, **P<0.01.

We have recently determined that cell-to-cell spread is a critical determinant of *S*. *flexneri* pathogenesis [28]. Importantly, the severity of epithelial fenestration (absence of luminal epithelium) and bloody diarrhea was contingent on efficient cell-to-cell spread. We therefore evaluated the impact of the observed cell-to-cell spreading defect in animals infected with the Δ*ipgD* strain on pathogenesis by measuring the level of epithelial fenestration and scoring dysentery (described in Materials and methods). In agreement with the role of cell-to-cell spread in ulceration, histopathological examination indicated that the Δ*ipgD* mutant caused significantly less epithelial fenestration (Fig 7A and 7B). In addition, animals infected with Δ*ipgD* bacteria exhibited significantly weaker bloody diarrheal symptoms than animals infected with wild type bacteria (Fig 7C and 7D). These results indicate that the Δ*ipgD* mutant is attenuated *in vivo*, and further establish the importance of cell-to-cell spread in pathogenesis.

## Discussion

Several intracellular pathogens manipulate the actin cytoskeleton to display actin-based motility and form membrane protrusions at cell-cell contacts that project into adjacent cells [22,42]. The existence of structures such as cortical actin at cell-cell contacts has been suggested to act as a barrier to dissemination [43,44]. *Listeria monocytogenes* has evolved specific mechanisms to counter global restriction through secretion of Internalin C, a bacterial factor that overcomes membrane tension at cell-cell contacts by interfering with TUBA and N-WASP interactions [44]. Similarly, *Rickettsia parkeri* facilitates protrusion formation through secretion of Sca4, a T4SS effector protein that mediates global reduction in actomyosin cortical tension [43]. Moreover, recent studies have shown that *Shigella flexneri* utilizes the T3SS translocase IpaC to decrease membrane tension [27]. Our results indicate that *S*. *flexneri* forms protrusions by piercing through cortical actin, deforming the plasma membrane, and forming a membrane bound compartment with low cortical actin levels (Fig 4). Our results also indicate that *de novo* cortical actin formation in protrusions represents a barrier to protrusion resolution and therefore emerges as a potential mechanism of restriction on the dissemination process (Fig 4 and S3 Movie). We note that local restriction on protrusion resolution is conceptually different from global restriction on protrusion formation through membrane tension, as previously suggested for *L*. *monocytogenes*, *R*. *parkeri* and *S*. *flexneri* [43,44].

Our results indicate that *S*. *flexneri* manipulates PtdIns(4,5)P_2_ levels to interfere with *de novo* cortical actin formation (Fig 3). It is likely that *de novo* cortical actin formation in protrusions may also restrict the dissemination of pathogens that spread through protrusion formation, including *L*. *monocytogenes*. Whether these pathogens resolve *de novo* cortical actin restriction through manipulation of PtdIns(4,5)P_2_ signaling remains to be determined. Interestingly, the resolution of *L*. *monocytogenes* protrusions requires the expression of the bacterial metalloprotease Mpl [45]. It is thus possible that *L*. *monocytogenes* resolves cytoskeleton restrictions in protrusions through direct degradation of components of the actin cytoskeleton, as opposed to manipulation of regulatory phosphoinositide signaling.

Our results indicate that *S*. *flexneri* utilizes the T3SS effector protein IpgD to manipulate PtdIns(4,5)P_2_ levels and cortical actin in membrane protrusions (Figs 3 and 4). The role of IpgD was first characterized in the context of studies focused on elucidating the mechanisms supporting the invasion process [31]. The phosphatidylinositol 4-phosphatase activity of IpgD was demonstrated *in vitro* and *in vivo* [29]. The connection between the activity of IpgD and the dynamics of the actin cytoskeleton was established through the observation that the ruffles formed at sites of entry appear smaller in cells infected with the *ipgD* mutant [30]. Moreover, when expressed in cells, IpgD lessens plasma membrane-cortical actin interactions, leading to morphological alterations in cells owing to reduced membrane tether forces [29]. In spite of these obvious connections to the dynamics of the actin cytoskeleton, IpgD is not required for efficient invasion in non-polarized cells [31], a result that we have confirmed in polarized cells (Fig 1B–1D) and in the colons of infant rabbits (Fig 6B). Thus, our results in the context of cell-to-cell spread provide an important demonstration of the functional importance of IpgD in cytoskeleton manipulation.

Our results show that *S*. *flexneri* expresses IpgD in order to hydrolyze PtdIns(4,5)P_2_ at the plasma membrane of the protrusions that project from primarily infected cells. Since PtdIns(4,5)P_2_ is displayed on the inner leaflet of the plasma membrane, this suggest that IpgD is secreted into the cytosolic compartment of protrusions, as opposed to translocated across the membrane. We note that the local secretion of IpgD in protrusions is consistent with the notion that the activity of the *S*. *flexneri* T3SS is differentially regulated depending on the cellular localization of the pathogen, with critical activation observed in protrusions and DMVs [46]. We have previously shown that the T3SS is required for protrusion resolution and DMV escape [18]. Although specific T3SS effector proteins, such as IcsB, support DMV escape [19,20], T3SS effector proteins that support protrusion resolution remained elusive. Thus, IpgD is the first example of a T3SS effector protein displaying function in protrusions (Fig 2) and not in DMVs (S3 Fig) during *S*. *flexneri* cell-to-cell spread. Our work therefore supports the notion that *S*. *flexneri* cell-to-cell spread is a multi-step process that is orchestrated in space and time by the T3SS [18,21].

Our results show that the role of IpgD in facilitating protrusion resolution has a significant impact on infection foci formation in tissue culture cells (Fig 1) and in the colon of infant rabbits (Fig 6A). Consistent with our previous work demonstrating the critical role of cell-to-cell spread in pathogenesis [28], the Δ*ipgD* mutant conferred milder symptoms of disease, including epithelial fenestration and bloody diarrhea (Fig 7). Our results therefore support the notion that inhibiting the activity of bacterial T3SS effector proteins supporting dissemination, such as IpgD, or modulating the host cell processes hijacked by these effector proteins, may alter the course of disease and may constitute therapeutic alternatives to antibiotic treatment [47].

## Materials and methods

### Ethics statement

All experiments described in this study were reviewed and approved by the University of Virginia Institutional Biosafety Committee and the Institutional Animal Care and Use Committee (protocol #4161).

### Bacterial strains, cell lines and growth conditions

The wild type (WT) *S*. *flexneri* strain used in this study is *S*. *flexneri* 2457T [6]. The Δ*ipgD* strain was generated by replacement of the coding region with a kanamycin cassette by homologous recombination, as previously described [48]. For complementation purposes, Δ*ipgD* was transformed with pACYC184-IpgD and pACYC184-IpgD^C438S^ kindly provided by Dr. Marcia Goldberg [32]. The pMMB207 plasmid harboring the gene encoding cyan fluorescent protein (pCFP) under the control of an isopropyl-β-D-thiogalactopyranoside (IPTG)-inducible promoter was introduced into *S*. *flexneri* strains by electroporation, resulting in WT pCFP and Δ*ipgD* pCFP. LB medium with appropriate antibiotics was used to culture *Escherichia coli* strains (DH5α and Δnic35) for cloning steps. Primers and generated constructs are listed in Tables A and B in S1 Text, respectively. Exponential phase *S*. *flexneri* strains cultured at 37°C were used for infecting cell monolayers. *S*. *flexneri* and *S*. *flexneri* pCFP glycerol stocks were re-streaked on LB agar containing 10 μg/ml Congo red dye (Fisher Chemical) supplemented with 10 μg/ml chloramphenicol when necessary and incubated at 37°C. The next day, a single Congo red positive colony of each strain was transferred to 2 ml LB (-/+ chloramphenicol) for overnight growth at 30°C. Overnight cultures were transferred to fresh LB (-/+ chloramphenicol) and cultured to exponential phase (37°C) to infect cell monolayers. Tryptic Soy Broth (TSB) supplemented with 10 μg/ml chloramphenicol was used to culture *S*. *flexneri* pCFP strains for 12–13 h at 37°C on a rotating wheel for animal infections. The growth of HT-29 cell lines was carried out in McCoy’s 5A medium (Gibco) containing 10% heat inactivated fetal bovine serum (hiFBS) (Invitrogen) at 37°C with 5% CO_2_. HEK 293 cells were cultured in DMEM (Gibco) supplemented with 10% hiFBS. Lentiviruses were produced from HEK 293 cells to generate HT-29 cell lines stably expressing either cyan or yellow fluorescence proteins targeted to the plasma membrane (mbCFP or mbYFP), mCherry fused to the PH domain of PLCδ (referred to as the mCherry-PH probe), or mCherry-fused to β-actin (mCherry-Actin), using packaging vectors pCMVΔ8.2Δvpr and pMD2.G [49]. The pMXsIP-mCherry vector was used for cloning; corresponding primers and plasmids are listed (Tables A and B in S1 Text), respectively.

### Infection of HT-29 cell monolayers and image analysis

Cell-to-cell spread phenotypes were tested by infecting confluent HT-29 cell monolayers in 96-well plates (Corning) with exponential phase *S*. *flexneri*. Following the delivery of *S*. *flexneri* inoculated media to each well, 96-well plates were centrifuged (1000 rpm, 5 min, room temperature) to initiate infection. Plates were incubated at 37°C with 5% CO_2_ for 1 h, then fresh McCoy’s medium supplemented with gentamicin was added to eliminate extracellular *S*. *flexneri* (final gentamicin concentration, 50 μg/ ml). Plates were incubated for 15 h before paraformaldehyde fixation (4%, 20 min, room temperature). Infection foci were detected by immunostaining using a primary unconjugated rabbit *S*. *flexneri* antibody (ViroStat, 1:1000, 90 min, room temperature) and secondary goat anti-rabbit IgG conjugated with Pacific blue (Invitrogen, 1:1000, 90 min, room temperature). Images were acquired with ImageXpress Micro imaging system (Molecular Devices). The size of each *S*. *flexneri* infection focus was measured with the Region function of the MetaMorph software. Three independent biological replicates were performed and 50 foci per strain were analyzed for each biological replicate. To assess invasion phenotype, foci count per image frame was determined using the same images from infection foci size experiments. As another measure for invasion, gentamicin protection assays were conducted in 96-well plates to determine the number of intracellular bacteria. Following the invasion period (37°C, 5% CO_2_, 1 h), the medium was replaced with gentamicin-supplemented medium (3 wells per strain). After 1 h in the presence of gentamicin, wells were washed with DPBS and cells were lysed in 0.1% triton-X on ice for 12 min. After lysis, serial dilutions were plated on LB Congo red plates and plates were incubated at 37°C overnight for colony forming unit determination.

### Live imaging

Time lapse confocal microscopy was applied to characterize the dynamics of *S*. *flexneri* pCFP spread, PtdIns(4,5)P_2_ and cortical actin at protrusion membranes in monolayers of HT-29 mbYFP, mixed populations of HT-29 mbYFP and HT-29 mbYFP/mCherry-PH (1:1), and mixed populations of HT-29 mbYFP and HT-29 mbYFP/mCherry-actin (1:1), respectively. Confluent monolayers of cells in 8-chamber coverglasses (Lab-TEK II, Thermo Fisher) were infected with *S*. *flexneri* pCFP. Coverglasses were centrifuged (800 rpm, 4 min, room temperature) to initiate invasion. After 1 h invasion period and inducing CFP expression with IPTG, live imaging was conducted every 2 min for 6 h on a Leica DMI 8 spinning-disc 474 confocal microscope controlled by the iQ software (Andor). Imaris software (Bitplane) was used for analysis. Three independent biological replicates were conducted and movies generated for *S*. *flexneri* spreading dynamics were analyzed according to Fig 2A. Movies generated for dynamics of PtdIns(4,5)P_2_ (mCherry-PH) levels and cortical actin (mCherry-Actin) levels at protrusion membranes were analyzed as described in Fig 3. Briefly, mCherry-PH and mCherry-Actin levels at protrusion membrane were reported as percentage of corresponding signal levels at membrane of mCherry-PH and mCherry-Actin expressing cells, respectively. Supporting movies (S1–S3 Movies) were generated using the Imaris software (Bitplane).

### Immunostaining

Co-localization of PtdIns(4,5)P_2_/F-actin and mCherry-Actin/F-actin were tested at 4 h post infection (hpi) in mixed HT-29 mbCFP and HT-29 mbCFP/mCherry-PH (1:1), and HT-29 mbCFP and HT-29 mbCFP/mCherry-Actin cells, respectively. Confluent cell monolayers were established on glass coverslips in 24-well plates and infected with overnight *S*. *flexneri* pCFP cultures (in McCoys’s-10% hiFBS). After 1 h of invasion and washing steps, McCoys’s-10% hiFBS supplemented with gentamicin and IPTG was delivered to each well to eliminate extracellular *S*. *flexneri* and to induce CFP expression, respectively. Following paraformaldehyde fixation, incubation with Phallodin-514 was performed for detection of F-actin. Formation of actin tails by *S*. *flexneri* pCFP strains in cells were visualized at 4 hpi in HT-29 cell monolayers after incubation with Alexa Fluor Phalloidin (either 514 or 594) (Invitrogen). Coverslips were mounted and images were acquired with a Leica DMI 8 spinning-disc 474 confocal microscope controlled by the iQ software (Andor). Protrusions projecting from probe-expressing cells (PH+ or Actin+) into probe-negative (PH- or Actin-) cells were evaluated as described in Fig 3.

### Infant rabbit infections

Pregnant New Zealand White Rabbits were obtained from the Charles River breeding company. Infant rabbits were handled as previously described [28]. Two days prior to infection, *S*. *flexneri* pCFP strains were re-streaked on LB-Congo red-chloramphenicol plates and incubated at 37°C for overnight. Congo red-positive colonies were transferred to 5 ml TSB-chloramphenicol and incubated as described in the first section of Materials and methods. On infection day, overnight cultures were pooled in 50 ml falcon tubes, centrifuged (4000 rpm /10 min / room temperature) and resuspended in TSB-chloramphenicol. Prior to infection, *S*. *flexneri* pCFP suspensions were centrifuged, bacterial pellets were resuspended in PBS and diluted 10-fold in PBS for rectal inoculation of 1.5x10^8^ cfus in anesthetized, 10–15 day old infant rabbits. Bacillary dysentery pathological scores were determined blindly. Dysentery scores were determined by presence (1) or absence (0) of bloody stain on the fur, and severity of diarrhea scores (wet body parts) were determined as (1) genitals only, (2) genitals and belly and (3) genitals, belly and legs. University of Virginia Institutional Biosafety Committee and the Institutional Animal Care and Use Committee reviewed and approved animal experiments. Care of the does and infant rabbits was executed according to standard operating procedures developed in coordination with the veterinary and animal care staff of the Center for Comparative Medicine at the University of Virginia.

### Histological examinations of infant rabbit colons

Animals infected with *S*. *flexneri* pCFP strains were euthanized by CO_2_ asphyxiation followed by euthasol injection. Bacterial invasion and cell death phenotypes were evaluated in colon samples collected 4 hpi. *S*. *flexneri* dissemination and infection-associated fenestration of colonic epithelium were evaluated in colon samples collected 8 hpi. Harvested animal colons were prepared for paraffin sections as previously described [28]. Colon paraffin sections were further processed for immunofluorescence or hematoxylin and eosin stains. To detect *S*. *flexneri* pCFP and epithelial cells, colon paraffin sections were sequentially processed via deparaffinization, re-hydration, antigen retrieval, permeabilization, and blocking as previously described [28]. The processed sections were incubated with rabbit anti *S*. *flexneri* antibody (ViroStat, 1:100) and mouse anti E-cadherin antibody (BD Biosciences, 1:100) at 4°C overnight in a humidified chamber. Sections were then incubated with goat anti-rabbit IgG conjugated with Pacific blue and (1:500) and Alexa Fluor goat 514 anti-mouse (1:500) secondary antibodies for 2 h at room temperature. Coverslips were mounted with ProLong Gold Antifade Mountant (Thermo Fisher). Cell death in colon sections were determined by using *in situ* cell death detection kit (Roche) along with DAPI staining. Entire colons (4 hpi) were imaged using a Nikon TE2000 microscope equipped for multi-color imaging including motorized stage and filter wheels and a Hamamatsu Orca ER Digital CCD Camera. Entire colons (8 hpi) were scanned for bacterial infection foci and imaged using a Nikon TE2000 microscope. Each image was processed with thresholding and masking functions of the MetaMorph software to detect intracellular *Shigella* that invaded E-cadherin positive epithelial cells. As described in S7 Fig, total infection foci count and average number of bacteria per focus were reported for a whole colon section (4 hpi; WT, *n = 6*; Δ*ipgD*, *n = 7*). The size of the infection foci was determined with images of 8 hpi colon sections (6 colons for each strain) using the Region function of the MetaMorph software (Molecular Devices, Inc.). Hematoxylin and eosin stains of colon sections were conducted at the Research Histology Core Facility at University of Virginia School of Medicine. H&E staining were scanned with an Aperio ScanScope Slide Scanner (Leica Biosystems), and analyzed to determine the amount of fenestration using the Aperio ImageScope software (Leica Biosystems). The percentage of fenestration for each colon was calculated as: (length of colon with fenestrated epithelium/ total length of colon) x 100.

### Statistical analyses

Analysis of a single variable across two groups was performed with unpaired t-test to determine statistical significance. Paired t-test was used to for the comparison of probe levels at different time points (nascent vs late) within the same protrusion. Experiments with multiple groups were analyzed with one-way ANOVA followed by Tukey’s multiple comparisons. Two-factor analysis was performed with two-way ANOVA followed by Sidak’s multiple comparisons. Two linear regression models were built to analyze data pertinent to co-localization at protrusion membranes: F-actin at protrusion membranes was explained by mCherry-PH probe recruitment, strain and their interaction or by mCherry-Actin recruitment, strain, and their interaction. Mixed model analysis was performed for the analysis of bacterial count per infection focus in colon sections explaining the natural log-transformed number of bacteria per infection focus using the genotype and a random intercept for each rabbit colon. P-values reported in this study are NS not significant, * < 0.05, ** < 0.01, *** < 0.001, and **** < 0.0001. GraphPad Prism 9.0.2 and R 4.1.1 were used for statistical analysis.

## Supporting information

S1 Fig *S*. *flexneri* pCFP spreading dynamics. Tracking data from independent biological replicate 2 (A) and 3 (B) showing WT and Δ*ipgD S*. *flexneri* pCFP cell-to-cell spreading dynamics. The length of the bars reflects the time spent in each color-coded compartment relative to Fig 1A.
(TIF)

S2 Fig ARP2/3 localization at bacterial pole inside protrusions. Representative images showing *S*. *flexneri* pCFP (blue, WT and Δ*ipgD)* in a protrusion (membrane, yellow) and Arp3 (red) localization at the bacterial pole. Graph showing Arp3 enrichment corresponding to signal intensities at the bacterial pole (white stars) normalized to signal intensities of local background. Circles represent data points and colors indicate independent biological replicate groups; bars indicate means of 90 measurements for each strain with s.d.; (*n* = 3). Scale bar is 2 μm.
(TIF)

S3 Fig Time spent in protrusion and DMV during *S*. *flexneri* spread. Quantification of time *S*. *flexneri* pCFP (WT and Δ*ipgD*) spent in DMVs before successful escape, protrusion resolution and protrusion collapse. Unpaired t-test shows no significant difference (NS).
(TIF)

S4 Fig Rescue of Δ*ipgD* protrusion resolution defect and correlation with PtdIns(4,5)P_2_ levels. (A) Graph showing the percentage of successful (red bar) or failed protrusion resolution (blue bar) by complementation strains. Bars represent means with s.d. for each outcome after analysis of 21 Δ*ipgD* pIpgD pCFP and 18 Δ*ipgD* pIpgD::C438S protrusions from four independent biological replicates. Statistics: two-way ANOVA statistical analysis followed by Sidak’s multiple comparison test comparing strains; *P< 0.05. (B) Graphs showing how mCherry-PH probe levels (%) change at nascent protrusion membrane and late protrusion membrane during successful protrusion resolution and protrusion collapse by Δ*ipgD* pIpgD pCFP and Δ*ipgD* pIpgD::C438S pCFP strains (PM, Protrusion Membrane; CM, Cell Membrane). Circles represent data points and colors indicate independent biological replicate groups; 21 mCherry-PH measurements at Δ*ipgD* pIpgD pCFP protrusions (nascent, late) and 10 mCherry-PH measurements at Δ*ipgD* pIpgD::C438S pCFP protrusions (nascent, late) for protrusion resolution; 8 mCherry-PH measurements at Δ*ipgD* pIpgD::C438S pCFP protrusions (nascent, late) for protrusion collapse; (*n* = 4). Statistics: paired t-test analysis; ****P<0.0001, *P<0.05; NS.
(TIF)

S5 Fig The mCherry-Actin probe reports on F-actin in during infection in HT-29 cells. Representative images showing co-localization of mCherry-Actin (red) with F-actin (yellow) at *S*. *flexneri* pCFP (blue) protrusion projecting from mCherry-Actin (+) cell to mCherry-Actin (-). Small white arrows indicate *S*. *flexneri* pCFP actin tail inside the protrusion; large white arrows indicate cortical actin underneath the protrusion membrane. Scale bar is 1 μm. Linear regression plot showing mCherry-Actin and F-actin signals measured in 15 WT pCFP protrusions (*n = 3* independent biological replicates) and 20 Δ*ipgD* pCFP protrusions (*n = 4* independent biological replicates). Quantification of signals were conducted as described in Fig 3. Black circles indicate measurements from WT pCFP protrusions; red squares indicate measurements from Δ*ipgD* pCFP protrusions. Significance of mCherry-Actin and F-actin relationship is determined by the difference of slope from zero. ****P<0.0001 based on a linear regression analysis explaining F-actin by mCherry-Actin, strain, and an interaction between mCherry-Actin and strain (R^2^ = 0.57, F_3,32_ = 14.35, P<0.0001). Slopes of WT pCFP and Δ*ipgD* pCFP are not statistically different (P = 0.289).
(TIF)

S6 Fig Quantification of mCherry-PH probe and F-actin levels at the plasma membrane surrounding bacteria in protrusions. (A) Representative images showing *S*. *flexneri* (*Shigella*, blue) in cells expressing membrane CFP (CM, Cell Membrane) forming protrusions that project from mCherry-PH(+) cells (red) into mCherry-PH(-) cells. (B) Images showing sites where mCherry-PH probe and F-actin signals were recorded using the Imaris software: white stars, protrusion; white diamonds, mCherry-PH(+) cell membrane; white squares, local background. (C) Example of calculation showing percentage of mCherry-PH probe and F-actin signal levels relative to respective signals at cell-cell contacts.
(TIF)

S7 Fig Quantification of number of invasion foci and number of bacteria per infection focus. (A) Panel i) Representative images of infant rabbit colon infected with *S*. *flexneri*. Red, *Shigella*; green, E-cadherin. Panel ii): Infection foci formed by *S*. *flexneri* (red) determined by computer-assisted analysis (see B for details below). Inset panel indicates quantified phenotypes (Parameter) and corresponding quantifications (Number). Bacteria per foci (bacteria / foci = 25) was calculated by dividing total bacterial count (706) with total invasion foci count (28). (B) Steps of computer-assisted identification of infection foci in colons section obtained from rabbits 4 hpi using metaXpress software: 1- defining colon site in the frame with white dashed lines 2- detecting colonic epithelial cells and *Shigella* via thresholding in the defined area 3- defining epithelium and bacteria via masking function. Computer detects co-localized regions in defined area as shown in upper A panel ii (intracellular *Shigella*). Scale bar is 30 μm.
(TIF)

S8 Fig Quantification of cell death in infected animal colons. (A) Representative images showing cell death (TUNEL+, blue, white arrowheads) at un-infected cells in colon sections (E-cadherin expressing cells, green) infected with *S*. *flexneri* pCFP (WT and Δ*ipgD*, red). Cell death indicated by white arrowheads at WT and Δ*ipgD* infected colons, small frames 1–4; rectangles, cell death occurring in un-infected cells. Graph showing total death count of un-infected cells in entire colon comprehensively imaged (6 colons for WT; 7 colons for Δ*ipgD*). Scale bar is 30 μm. NS, not significant based on unpaired t-test. (B) Representative images showing cell death (TUNEL+, blue, white arrowheads) at infected cells in colon sections (E-cadherin expressing cells, green) infected with *S*. *flexneri* pCFP (WT and Δ*ipgD*, red). Cell death indicated by white arrowheads at WT and Δ*ipgD* infected colons, small frames 1–4; rectangles, cell death occurring in infected cells. Graph showing total death count of infected cells in entire colon comprehensively imaged (6 colons for WT; 7 colons for Δ*ipgD*). Scale bar is 30 μm. NS, not significant based on unpaired t-test.
(TIF)

S1 Text Table A in S1 Text. Primers used in this study. Table B in S1 Text. Plasmid constructs used in this study.
(DOCX)

S1 Movie Successful *Shigella* dissemination in colonic epithelial cells. HT-29 mbYFP (yellow) cells were infected *S*. *flexneri* WT pCFP (blue) for 6 h. This movie shows the formation of canonical membrane compartments and escape from a DMV during *Shigella* dissemination. Images were acquired every 2 min. This movie corresponds to the successful spread by the WT pCFP strain shown in Fig 2B.
(MP4)

S2 Movie A failed *Shigella* dissemination in colonic epithelial cells. HT-29 mbYFP (yellow) cells were infected *S*. *flexneri* Δ*ipgD* pCFP (blue) for 6 h. This movie shows that Δ*ipgD* pCFP strain forms a protrusion yet cannot form VLP and eventually collapses back to the primarily infected cell. Images were acquired every 2 min.
(MP4)

S3 Movie A failed Δ*ipgD* dissemination in which actin accumulation occurs at protrusion membrane. Initially, a blue Δ*ipgD* pCFP forms an actin-negative protrusion projecting from HT-29 mbYFP Cherry-Actin (+) cell to HT-29 mbYFP cell. However, cortical actin starts to accumulate at the protrusion membrane which coincides with the pause in elongation. Eventually, the protrusion enriched with polymerized cortical actin collapses back to the primarily infected cell. Images were acquired every 2 min.
(MP4)
