# Supplementary figures and images for: The type 3 secretion effector IpgD promotes S. flexneri dissemination
Source: PLoS Pathog. 2022 Feb 7;18(2):e1010324. doi: 10.1371/journal.ppat.1010324 (PMC8853559; doi:10.1371/journal.ppat.1010324)

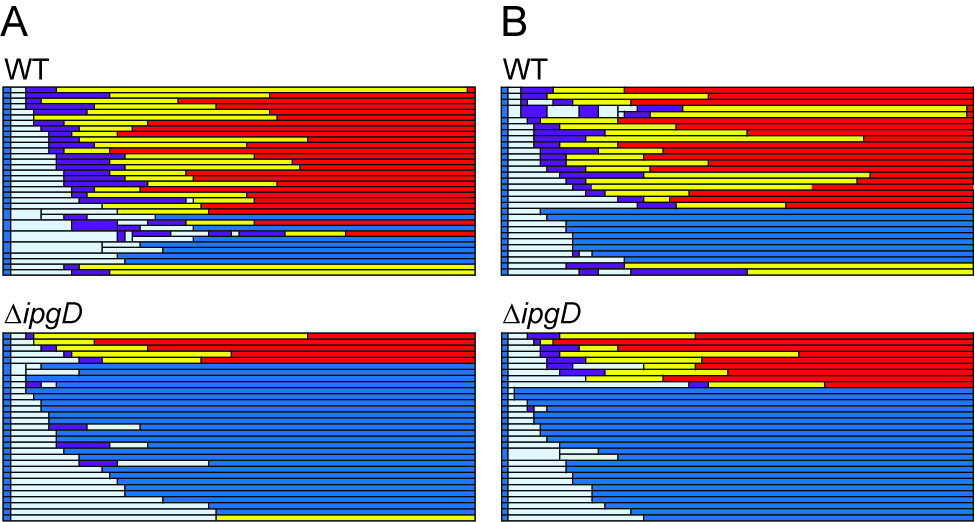

Supplement: S1 Fig — Tracking data from independent biological replicate 2 (A) and 3 (B) showing WT and ΔipgD S. flexneri pCFP cell-to-cell spreading dynamics. The length of the bars reflects the time spent in each color-coded compartment relative to Fig 1A. (TIF) [file ppat.1010324.s001.tif]

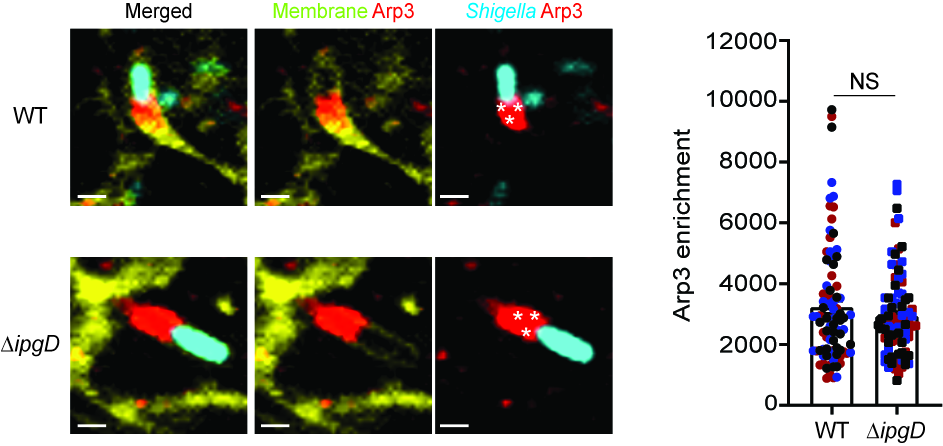

Supplement: S2 Fig — Representative images showing S. flexneri pCFP (blue, WT and ΔipgD) in a protrusion (membrane, yellow) and Arp3 (red) localization at the bacterial pole. Graph showing Arp3 enrichment corresponding to signal intensities at the bacterial pole (white stars) normalized to signal intensities of local background. Circles represent data points and colors indicate independent biological replicate groups; bars indicate means of 90 measurements for each strain with s.d.; (n = 3). Scale bar is 2 μm. (TIF) [file ppat.1010324.s002.tif]

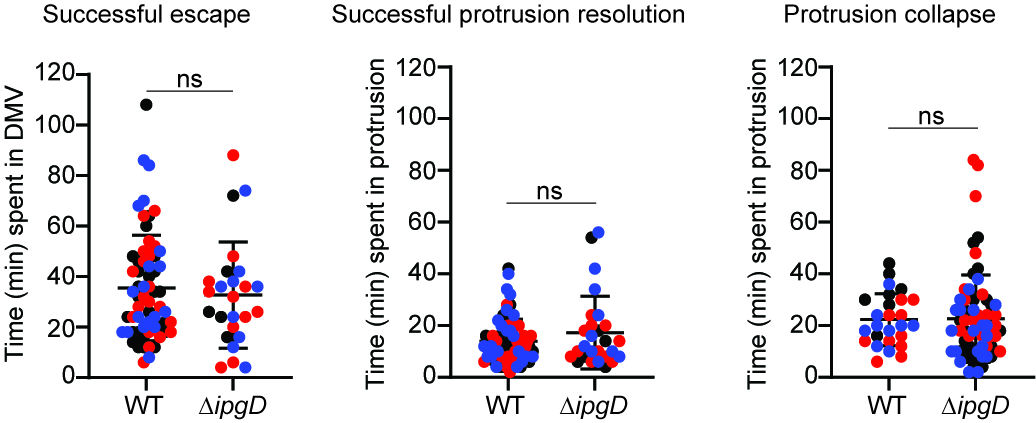

Supplement: S3 Fig — Quantification of time S. flexneri pCFP (WT and ΔipgD) spent in DMVs before successful escape, protrusion resolution and protrusion collapse. Unpaired t-test shows no significant difference (NS). (TIF) [file ppat.1010324.s003.tif]

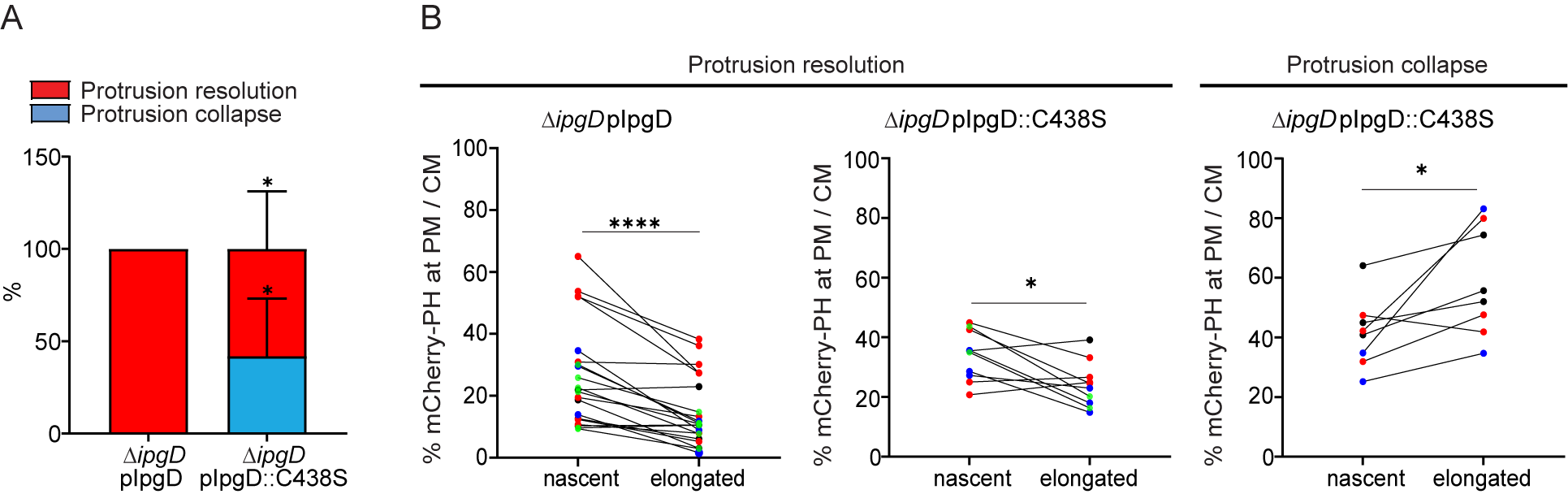

Supplement: S4 Fig — (A) Graph showing the percentage of successful (red bar) or failed protrusion resolution (blue bar) by complementation strains. Bars represent means with s.d. for each outcome after analysis of 21 ΔipgD pIpgD pCFP and 18 ΔipgD pIpgD::C438S protrusions from four independent biological replicates. Statistics: two-way ANOVA statistical analysis followed by Sidak’s multiple comparison test comparing strains; *P< 0.05. (B) Graphs showing how mCherry-PH probe levels (%) change at nascent protrusion membrane and late protrusion membrane during successful protrusion resolution and protrusion collapse by ΔipgD pIpgD pCFP and ΔipgD pIpgD::C438S pCFP strains (PM, Protrusion Membrane; CM, Cell Membrane). Circles represent data points and colors indicate independent biological replicate groups; 21 mCherry-PH measurements at ΔipgD pIpgD pCFP protrusions (nascent, late) and 10 mCherry-PH measurements at ΔipgD pIpgD::C438S pCFP protrusions (nascent, late) for protrusion resolution; 8 mCherry-PH measurements at ΔipgD pIpgD::C438S pCFP protrusions (nascent, late) for protrusion collapse; (n = 4). Statistics: paired t-test analysis; ****P<0.0001, *P<0.05; NS. (TIF) [file ppat.1010324.s004.tif]

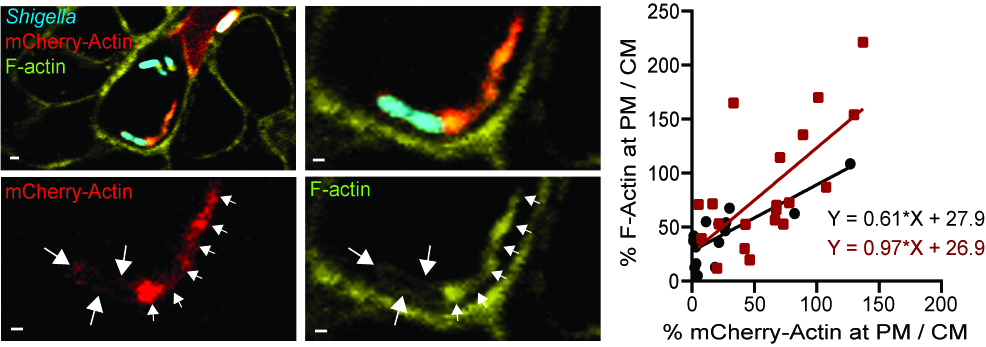

Supplement: S5 Fig — Representative images showing co-localization of mCherry-Actin (red) with F-actin (yellow) at S. flexneri pCFP (blue) protrusion projecting from mCherry-Actin (+) cell to mCherry-Actin (-). Small white arrows indicate S. flexneri pCFP actin tail inside the protrusion; large white arrows indicate cortical actin underneath the protrusion membrane. Scale bar is 1 μm. Linear regression plot showing mCherry-Actin and F-actin signals measured in 15 WT pCFP protrusions (n = 3 independent biological replicates) and 20 ΔipgD pCFP protrusions (n = 4 independent biological replicates). Quantification of signals were conducted as described in Fig 3. Black circles indicate measurements from WT pCFP protrusions; red squares indicate measurements from ΔipgD pCFP protrusions. Significance of mCherry-Actin and F-actin relationship is determined by the difference of slope from zero. ****P<0.0001 based on a linear regression analysis explaining F-actin by mCherry-Actin, strain, and an interaction between mCherry-Actin and strain (R2 = 0.57, F3,32 = 14.35, P<0.0001). Slopes of WT pCFP and ΔipgD pCFP are not statistically different (P = 0.289). (TIF) [file ppat.1010324.s005.tif]

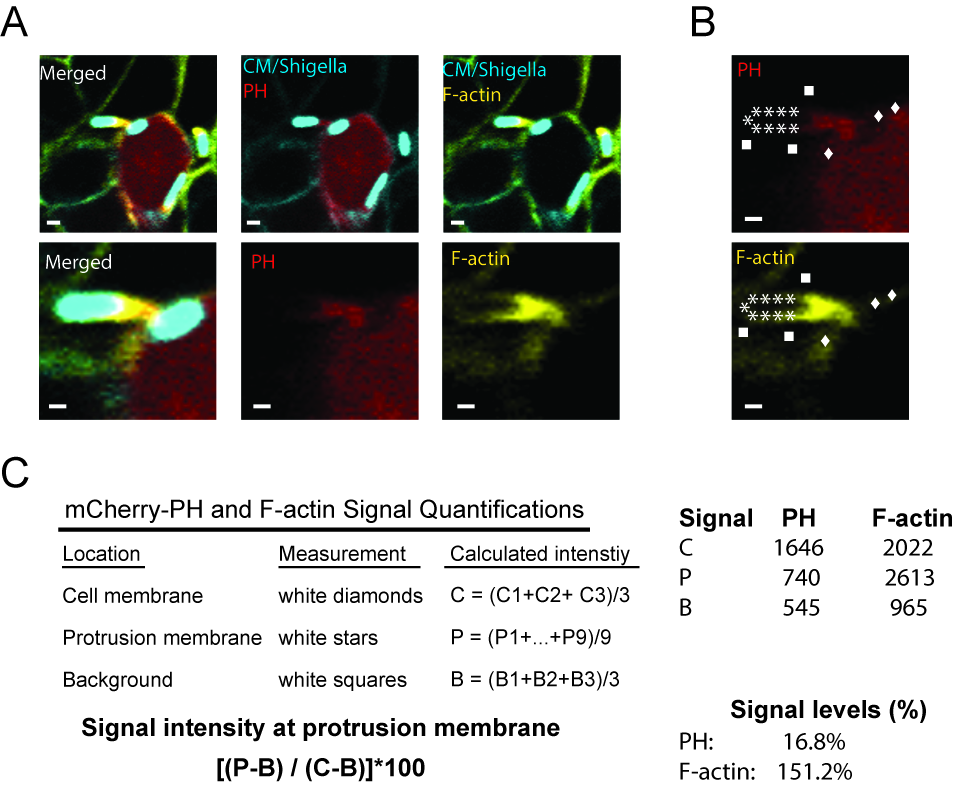

Supplement: S6 Fig — (A) Representative images showing S. flexneri (Shigella, blue) in cells expressing membrane CFP (CM, Cell Membrane) forming protrusions that project from mCherry-PH(+) cells (red) into mCherry-PH(-) cells. (B) Images showing sites where mCherry-PH probe and F-actin signals were recorded using the Imaris software: white stars, protrusion; white diamonds, mCherry-PH(+) cell membrane; white squares, local background. (C) Example of calculation showing percentage of mCherry-PH probe and F-actin signal levels relative to respective signals at cell-cell contacts. (TIF) [file ppat.1010324.s006.tif]

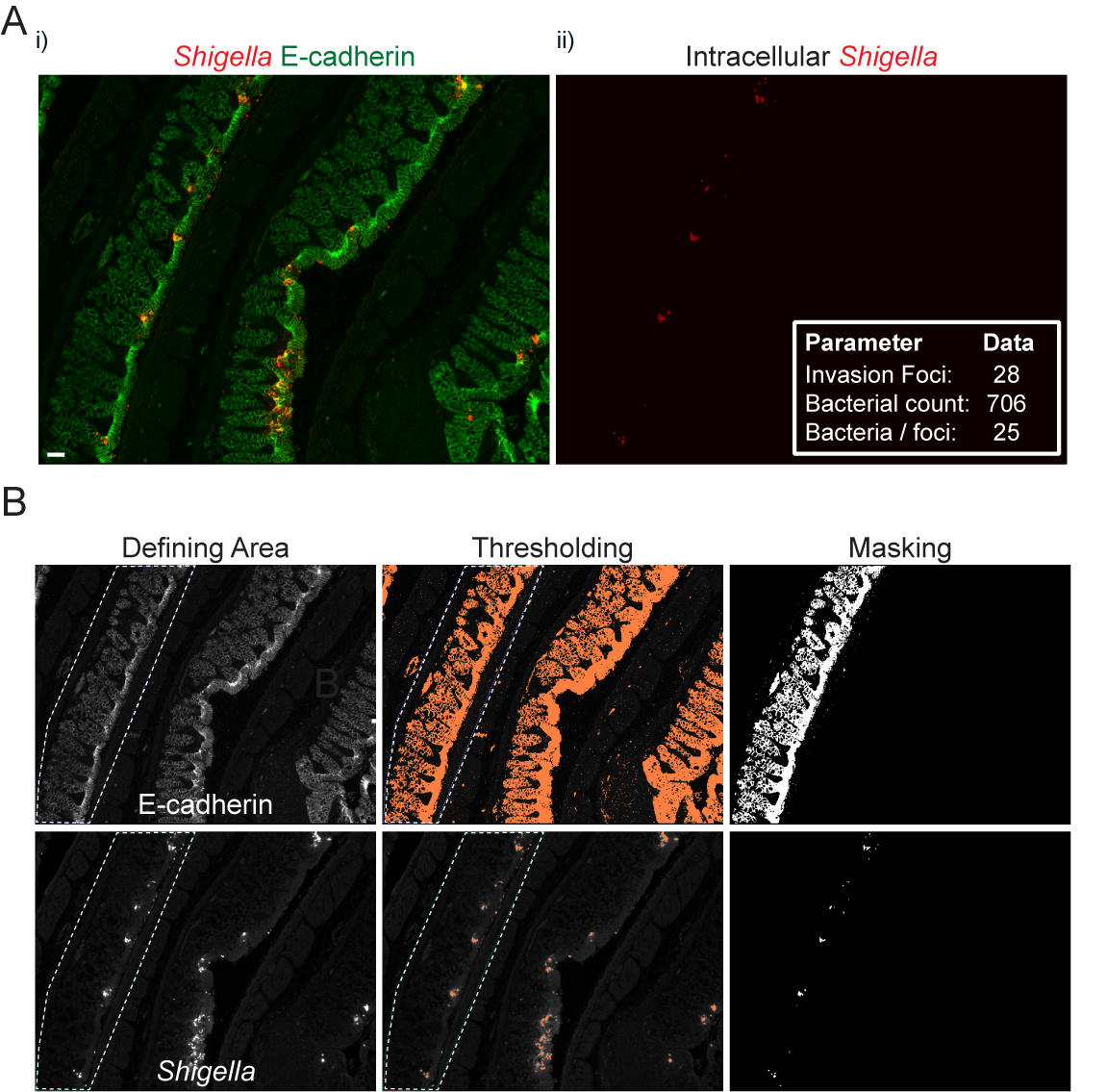

Supplement: S7 Fig — (A) Panel i) Representative images of infant rabbit colon infected with S. flexneri. Red, Shigella; green, E-cadherin. Panel ii): Infection foci formed by S. flexneri (red) determined by computer-assisted analysis (see B for details below). Inset panel indicates quantified phenotypes (Parameter) and corresponding quantifications (Number). Bacteria per foci (bacteria / foci = 25) was calculated by dividing total bacterial count (706) with total invasion foci count (28). (B) Steps of computer-assisted identification of infection foci in colons section obtained from rabbits 4 hpi using metaXpress software: 1- defining colon site in the frame with white dashed lines 2- detecting colonic epithelial cells and Shigella via thresholding in the defined area 3- defining epithelium and bacteria via masking function. Computer detects co-localized regions in defined area as shown in upper A panel ii (intracellular Shigella). Scale bar is 30 μm. (TIF) [file ppat.1010324.s007.tif]

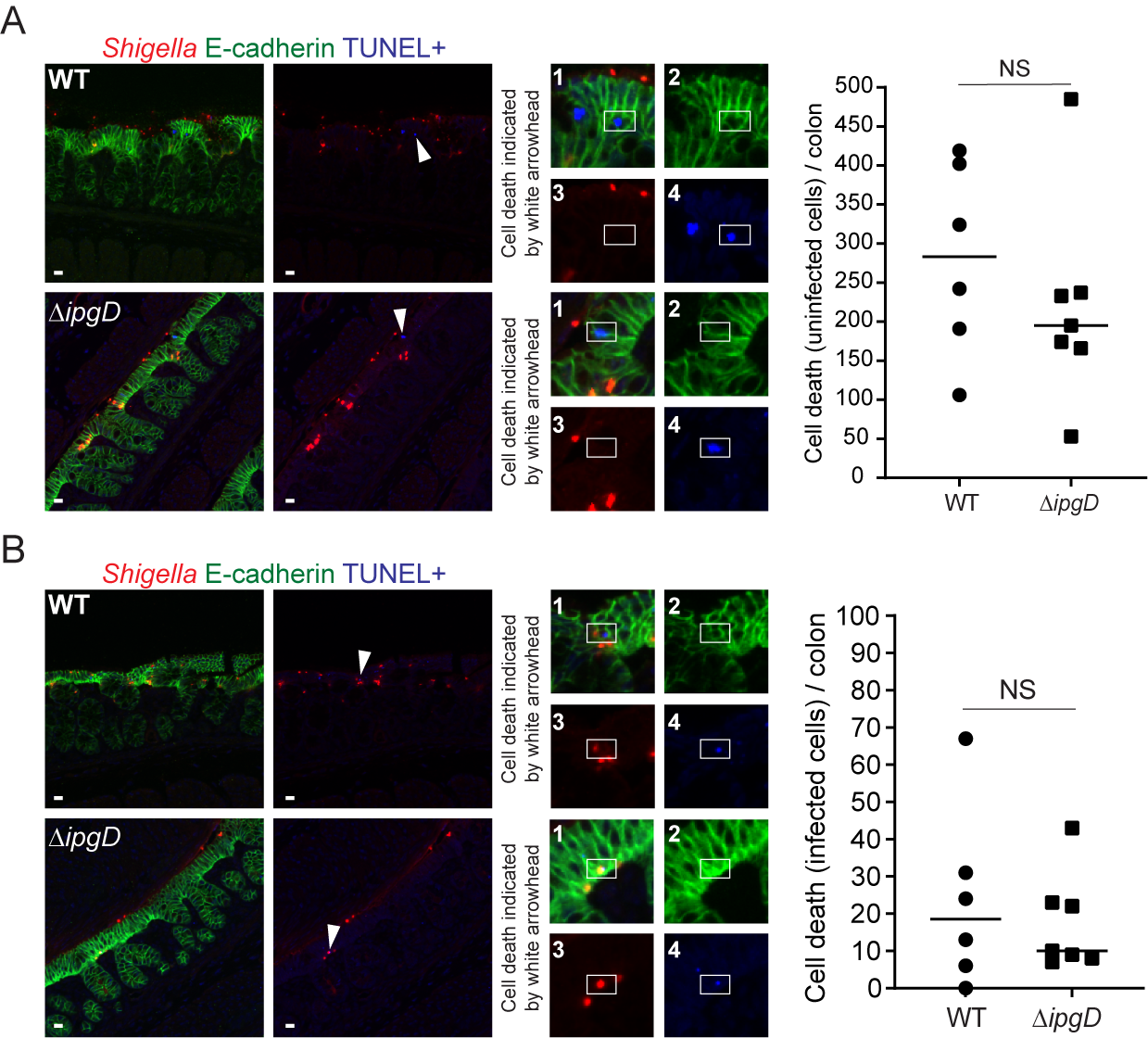

Supplement: S8 Fig — (A) Representative images showing cell death (TUNEL+, blue, white arrowheads) at un-infected cells in colon sections (E-cadherin expressing cells, green) infected with S. flexneri pCFP (WT and ΔipgD, red). Cell death indicated by white arrowheads at WT and ΔipgD infected colons, small frames 1–4; rectangles, cell death occurring in un-infected cells. Graph showing total death count of un-infected cells in entire colon comprehensively imaged (6 colons for WT; 7 colons for ΔipgD). Scale bar is 30 μm. NS, not significant based on unpaired t-test. (B) Representative images showing cell death (TUNEL+, blue, white arrowheads) at infected cells in colon sections (E-cadherin expressing cells, green) infected with S. flexneri pCFP (WT and ΔipgD, red). Cell death indicated by white arrowheads at WT and ΔipgD infected colons, small frames 1–4; rectangles, cell death occurring in infected cells. Graph showing total death count of infected cells in entire colon comprehensively imaged (6 colons for WT; 7 colons for ΔipgD). Scale bar is 30 μm. NS, not significant based on unpaired t-test. (TIF) [file ppat.1010324.s008.tif]
