# Supplementary material for: The type 3 secretion effector IpgD promotes S. flexneri dissemination
Source: PLoS Pathog. 2022 Feb 7;18(2):e1010324. doi: 10.1371/journal.ppat.1010324 (PMC8853559; doi:10.1371/journal.ppat.1010324)
Supplement: S1 Text — Primers used in this study. Table B in S1 Text. Plasmid constructs used in this study. (DOCX) [file ppat.1010324.s009.docx]

**Table A. Primers used in this study**

| **Purpose** | **Primers** |
| --- | --- |
| *ipgD* knock-out | 5ipgd>km: ctctgcggtattcaacattaatttg |
|  | 3ipgd>km: cattataccataatctttacttcc |
|  | 5ipgd-km: catttatgactgaggataattaaatggctaaaatgagaatatcaccggaattg |
|  | 3ipgd-km: caattccggtgatattctcattttagccatttaattatcctcagtcataaatg |
|  | 5km-ipgd: attatattttactggatgaattgttttaagaggaatatatggaagatttagc |
|  | 3km-ipgd: gctaaatcttccatatattcctcttaaaacaattcatccagtaaaatataat |
| *ipgD* knock-out verification | 5ipgD: cctaaatgggttatgcctctatttc |
|  | 3ipgD: cataccgcatcattttgtgtttcgg |
| Stable PH expression | 5PH XhoI :aattctcgagggcacgggctccaggatgaccc |
|  | 3PH NotI: aattgcggccgccttctgccgctggtccatgga |
| Stable β-Actin expression | 5actin_XhoI: aattctcgagctggatccggactcagatcacgagct |
|  | 3actin_NotI: gcggccgcctagaagcatttgcggtggacg |

**Table B. Plasmid constructs used in this study**

| **Plasmid** | **Description** | **Source** |
| --- | --- | --- |
| pCFP | IPTG-inducible CFP expression vector for *Shigella* strains, chloramphenicol resistance | [1] |
| pCFP | IPTG-inducible CFP expression vector for *Shigella* strains, spectinomycin resistance | Erin A. Weddle |
| pSB890-*ipgD-kanR* | Vector harboring deletion fragment (*ipgD::kanR*) to generate Δ*ipgD* strain, tetracycline resistance | This study |
| pACYC184 IPGD-IPGE | Construct harboring genes that encode IpgD and its chaperon IpgE for complementation of to Δ*ipgD* strain | Marcia Goldberg |
| pACYC184 IPGD-C438S-IPGE | Construct harboring genes that encode mutated IpgD (Cysteine438 residue was replaced with Serine) and its chaperon IpgE for complementation of to Δ*ipgD* strain | Marcia Goldberg |
| pMX-mbYFP | Construct for constitutive YFP expression at the plasma membrane of HT-29 cells | [2] |
| pMX-PH | Construct for constitutive expression of PtdIns(4,5)P_2_ specific pleckstin homology (PH) domain of phospholipase C delta expression at the plasma membrane of HT-29 cells | This study |
| pMX-mbCFP | Construct for constitutive CFP expression at the plasma membrane of HT-29 cells | [2] |

**Reference**

1. Weddle E, Agaisse H. Spatial, Temporal, and Functional Assessment of LC3-Dependent Autophagy in Shigella flexneri Dissemination. Infect Immun. 2018;86(8). doi: 10.1128/IAI.00134-18. PubMed PMID: 29844234; PubMed Central PMCID: PMCPMC6056868.

2. Dragoi AM, Agaisse H. The class II phosphatidylinositol 3-phosphate kinase PIK3C2A promotes Shigella flexneri dissemination through formation of vacuole-like protrusions. Infect Immun. 2015;83(4):1695-704. doi: 10.1128/IAI.03138-14. PubMed PMID: 25667265; PubMed Central PMCID: PMCPMC4363405.
